# Early‐life stasis in partial seasonal migration is underpinned by among‐cohort variation in migratory plasticity and selective disappearance

**DOI:** 10.1111/1365-2656.70215

**Published:** 2026-01-28

**Authors:** Cassandra R. Ugland, Paul Acker, Sarah J. Burthe, Rita Fortuna, Carrie Gunn, Michael P. Harris, Josie H. Hewitt, Timothy I. Morley, Mark A. Newell, Robert L. Swann, Erin A. Taylor, Sarah Wanless, Francis Daunt, Jane M. Reid

**Affiliations:** ^1^ Department of Biology Norwegian University of Science and Technology Trondheim Norway; ^2^ UK Centre for Ecology & Hydrology Edinburgh UK; ^3^ School of Biological Sciences University of Aberdeen Aberdeen UK; ^4^ Highland Ringing Group Tain UK

**Keywords:** age‐specific life‐history, behavioural repeatability, cohort effects, multi‐state capture‐mark‐recapture, natural selection, partial migration, phenotypic plasticity, sub‐adult survival

## Abstract

1. Life‐history traits expressed in early life can exhibit considerable among‐cohort variation, which could substantially affect population age‐structure and dynamics if initial variation persists into later life‐stages. Yet, initial among‐cohort variation could be reinforced, rapidly dissipated, or else completely reshaped by dynamic combinations of age‐specific phenotypic plasticity and selective disappearance acting within and among cohorts. However, such effects have not been comprehensively quantified for any trait, precluding full prediction of the form and implications of phenotypic dynamics, and emerging age‐specific life‐history variation, in varying environments.
2. We provide a framework for conceptualising phenotypic change resulting from joint and interacting effects of cohort‐specific and age‐specific plasticity and selective disappearance. We implement this framework by quantifying overall early‐life age‐specific phenotypic change (or stasis), and dissecting underlying dynamics of plasticity and selection, for the ecologically critical life‐history trait of seasonal migration versus residence. We achieve this by fitting multi‐state models to extensive multi‐year ring‐resighting data from 9358 colour‐ringed European shags (*Gulosus aristotelis*) from 11 cohorts in a partially migratory population.
3. The overall cross‐cohort mean proportion of migrants versus residents remained approximately constant across the four winters following fledging, implying no overall change in the degree of seasonal migration with age. This stasis was underlain by consistently high cross‐year individual phenotypic repeatability, and by average plasticity towards residence that was counter‐acted by average selective disappearance of sub‐adult residents.
4. However, these cross‐cohort means obscured substantial among‐cohort variation in the initial degree of partial migration, and in subsequent joint effects of plasticity and selective disappearance. Here, plasticity and selection were not systematically associated within or across cohorts or ages, but rather reinforced versus counter‐acted each other at different times, thereby reshaping the pattern of among‐cohort variation in partial migration across ages.
5. These results demonstrate that an absence of overall age‐specific change in a key life‐history trait, seasonal migration versus residence, obscures substantial underlying variation in both early‐life plasticity and selective disappearance, generating complex phenotypic dynamics within individual cohorts. Standard cross‐cohort analyses may therefore inadequately predict future spatio‐seasonal dynamics, since novel age‐specific life‐histories could readily emerge given changing environmental drivers of plasticity and selection.

## Graphical abstract

Changes to mean early‐life phenotypes are fundamentally driven by joint dynamics of plasticity and selection, but such effects are rarely quantified. We show that cross‐cohort stasis in the degree of partial migration is underpinned by substantial within‐ and among‐cohort variation in plasticity and selection on migration, indicating high environmental sensitivity.

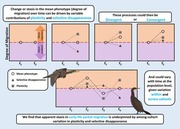

## 1 INTRODUCTION

The extent to which early‐life expression of life‐history traits varies among cohorts, and the subsequent extent to which initial among‐cohort variation persists into later life, could substantially shape overall population demography and dynamics (Beckerman et al., 2003; Forsythe et al., 2024; Lindström, 1999; Lindström & Kokko, 2002; Payo‐Payo et al., 2023). Persistent among‐cohort variation implies lagged environmental effects, where long‐lasting impacts of early‐life conditions could generate suboptimal subsequent phenotypes given temporally varying environmental conditions (Beckerman et al., 2003; Descamps et al., 2008; Lindström & Kokko, 2002). Any selection for or against specific phenotypes could then effectively act on specific age classes, altering population age‐structure (Payo‐Payo et al., 2023). Alternatively, initial among‐cohort variation could rapidly dissipate, if cohort‐specific development of life‐histories means that all cohorts converge towards the same phenotypic mean. Fully understanding and predicting emerging population age‐structure, demography and dynamics therefore requires quantifying initial among‐cohort variation in life‐history traits, and quantifying relationships with subsequent variation in the key processes that shape within‐ and among‐cohort changes across successive ages (Lindström, 1999; Lindström & Kokko, 2002). Yet, such relationships, and their underlying dynamics, are rarely fully conceptualized or quantified in any system.

In general, changing phenotypic means across ages following initial trait expression fundamentally result from two processes: labile phenotypic plasticity and selective disappearance (Supporting Information S1; van de Pol et al., 2012; van de Pol & Verhulst, 2006). Specifically, labile plasticity allows individuals to flexibly (and potentially reversibly) alter their phenotypes in response to changing environmental conditions following initial development. Systematic plasticity expressed within cohorts could therefore increase, decrease, or completely reshape initial among‐cohort variation, depending on how strongly plastic changes are positively or negatively correlated with initial phenotypes (Forsythe et al., 2024; Reid & Acker, 2022). Meanwhile, given some degree of individual phenotypic repeatability across years, selective disappearance (i.e. differential survival with respect to phenotype) could reduce the frequencies of specific phenotypes within a cohort, thereby further altering cohort means and among‐cohort variances across ages. These non‐exclusive processes of plasticity, repeatability and selection could interact to jointly increase or decrease initial among‐cohort variation (illustrated in Supporting Information S1). Indeed, while changes in cohort mean phenotypes across ages imply non‐zero underlying plasticity and/or selection, an absence of any change (i.e. phenotypic stasis) does not imply that neither process is acting, since their phenotypic effects could be opposing and hence cancel out (van de Pol et al., 2012; van de Pol & Verhulst, 2006).

Furthermore, given varying environmental conditions, the forms and magnitudes of plasticity and selection could vary among years and ages. This implies that different cohorts will experience differing drivers of phenotypic change at different times (Supporting Information S1; Lindström, 1999). Comprehensive dissection of life‐history variation and resulting population structure and dynamics therefore requires quantifying among‐ and within‐cohort variation in the forms and magnitudes of plasticity and selective disappearance with respect to focal life‐history traits and quantifying their joint effects. However, such dynamics and net impacts have not yet been comprehensively quantified for any trait. Rather, studies that quantify plasticity and/or selective disappearance in the context of age‐specific life‐history development commonly (implicitly) assume that both processes are temporally constant, and hence have consistent effects across cohorts and ages. Such studies could consequently miss biologically important dimensions of within‐ and among‐cohort variation (Forsythe et al., 2024; van de Pol & Verhulst, 2006).

One key life‐history trait that directly shapes population dynamics in spatially and seasonally varying environments is facultative seasonal migration versus year‐round residence. In many partially migratory taxa (including fish, mammals, birds and amphibians: Berg et al., 2019; Buchan et al., 2020; Chapman et al., 2012; Gillis et al., 2008; Gowan et al., 2019; Grayson et al., 2011), individuals sequentially express migration or residence through each non‐breeding season from early life. While these phenotypes are frequently highly repeatable in adults, early‐life plasticity often occurs (Berg et al., 2019; Xu et al., 2021). This plasticity can gradually reshape individual migration occurrence, timing and route (Campioni et al., 2020; Sergio et al., 2014; Wynn et al., 2022), commonly culminating in reduced migration with increasing age (Chan et al., 2024; Gowan et al., 2019; Witczak et al., 2024; but see Eggeman et al., 2016). Further, migrants and residents from the same breeding population can experience very different non‐breeding season environments, causing episodes of survival selection that can vary among years and ages and hence cohorts (Acker, Daunt, et al., 2021; Buchan et al., 2020; Ugland et al., 2024). Accordingly, there is clear potential for temporally varying reinforcing or counter‐acting effects of labile plasticity and selective disappearance to shape the forms and magnitudes of within‐ and among‐cohort variation in migration versus residence across successive ages. Such changes could in turn generate structured population‐level changes in the degree of seasonal movement (Sergio et al., 2014; Wynn et al., 2025). However, despite increasing interest in early‐life dynamics of seasonal migration and resulting potential for non‐breeding season range shifts, no studies have yet explicitly quantified early‐life among‐cohort variation in the degree of partial migration, nor quantified how such variation is reshaped across successive years through dynamic combinations of plasticity and selective disappearance.

These multifaceted objectives require quantifying age‐specific expression of seasonal migration versus residence, and associated survival versus mortality, of large numbers of individuals encompassing multiple cohorts across sequential non‐breeding seasons through early life. To achieve this, we fitted multi‐state capture‐mark‐recapture (MS‐CMR) models to large‐scale year‐round resightings of 9358 colour‐ringed European shags (*Gulosus aristotelis*, hereafter ‘shags’) fledged in 11 cohorts within a partially migratory population to elucidate five key components of early‐life expression, plasticity and selective disappearance. First, we quantified within‐ and among‐cohort variation in early‐life partial migration by quantifying cohort‐specific proportions of surviving individuals that were migrant versus resident in each of the four winters following fledging. We thereby quantified the degree to which the occurrence of seasonal migration changed systematically with age to adulthood, and whether such changes varied among cohorts. Second, we quantified the net magnitude of early‐life plasticity (i.e. phenotypic change) versus repeatability within cohorts between winters, and partitioned this plasticity into resident‐to‐migrant versus migrant‐to‐resident phenotypic transitions. Third, we explicitly tested for compensatory or reinforcing plasticity by quantifying the associations between initial among‐cohort variation in partial migration and the probabilities of subsequent resident‐to‐migrant and migrant‐to‐resident transitions and resulting partial migration. Fourth, we quantified the dynamics of early‐life survival selection on migration versus residence, and hence quantified among‐cohort variation in the magnitude and direction of age‐specific selective disappearance. Fifth, we explicitly quantified the among‐cohort variation in the contributions of age‐specific plasticity and selective disappearance to phenotypic changes in the degree of early‐life partial migration, and tested whether effects of plasticity and selective disappearance systematically reinforced versus counter‐acted each other. Together, these analyses provide a complete decomposition of overall early‐life phenotypic change evident across cohorts into the underlying causal dynamic processes of plasticity and selection acting within cohorts. We thereby reveal how within‐cohort processes can interact to reshape patterns of among‐cohort variation in a key life‐history trait, seasonal migration versus residence, and thereby fundamentally shape age‐specific spatio‐seasonal population dynamics.

## 2 METHODS

### 2.1 Study system

Our field system provides a highly relevant and tractable opportunity to quantify relative impacts of plasticity and selective disappearance on among‐cohort variation in early‐life partial migration, which has not previously been achieved. Shags breed in large colonies where numerous chicks from annual cohorts can be marked with field‐readable colour‐rings before fledging. Since all individuals return to land daily to dry and thermoregulate, colour‐ringed individuals can be resighted at coastal roosts throughout the year. This allows direct field observation of individuals' non‐breeding season locations and hence current status as resident or migrant, and inference on location‐dependent (and hence phenotype‐dependent) survival (Acker, Daunt, et al., 2021; Grist et al., 2014). Since shags predominantly first breed aged 3–4 years (range 2–6; Supporting Information S2), there is substantial opportunity for among‐cohort variation in plasticity and selection to reshape cohort‐specific degrees of partial migration through the winters preceding and spanning typical recruitment.

Accordingly, we implemented intensive colour‐ringing and year‐round resighting of shags fledged in a partially migratory population breeding on the Isle of May National Nature Reserve (hereafter ‘IoM’), Scotland (56°11′5.40″ N, 2°33′16.19″ W). Comprehensive reproductive monitoring and ringing during the 2010–2020 breeding seasons (April‐early August) generated 9358 colour‐ringed chicks spanning 11 cohorts (mean ± standard deviation: 851 ± 188 chicks/year, range 551–1125, all annual totals in Supporting Information S2). Through each non‐breeding season (August–February) spanning 2010–2024, we undertook regular (minimum fortnightly) intensive resighting surveys of known major shag roosts along the eastern Scottish coast, including IoM (Supporting Information S2). Additional less intensive surveys, including ‘citizen science’ contributions, provided some resightings from geographically wider sites (Acker, Daunt, et al., 2021; Grist et al., 2014, 2017). This effort generated a total of 25,076 non‐breeding season resightings of surviving individuals between fledging and their fourth winter, spanning the population's overall geographical range. Previous large‐scale surveys of all UK east coast breeding colonies demonstrated very high reproductive philopatry: 90% of recruiting individuals that originally fledged on IoM also bred on IoM, and most observed dispersing individuals bred <50 km away (Barlow et al., 2013). This implies that permanent dispersal outside the observed range is likely to be extremely rare, and that relatively long distance non‐breeding season movements in early life can be interpreted as migration rather than impending dispersal. Ringing and fieldwork activities were licensed by the British Trust for Ornithology and NatureScot (permits A400 and A4607). No further ethical approval was required.

### 2.2 Model structure

**FIGURE 1:**
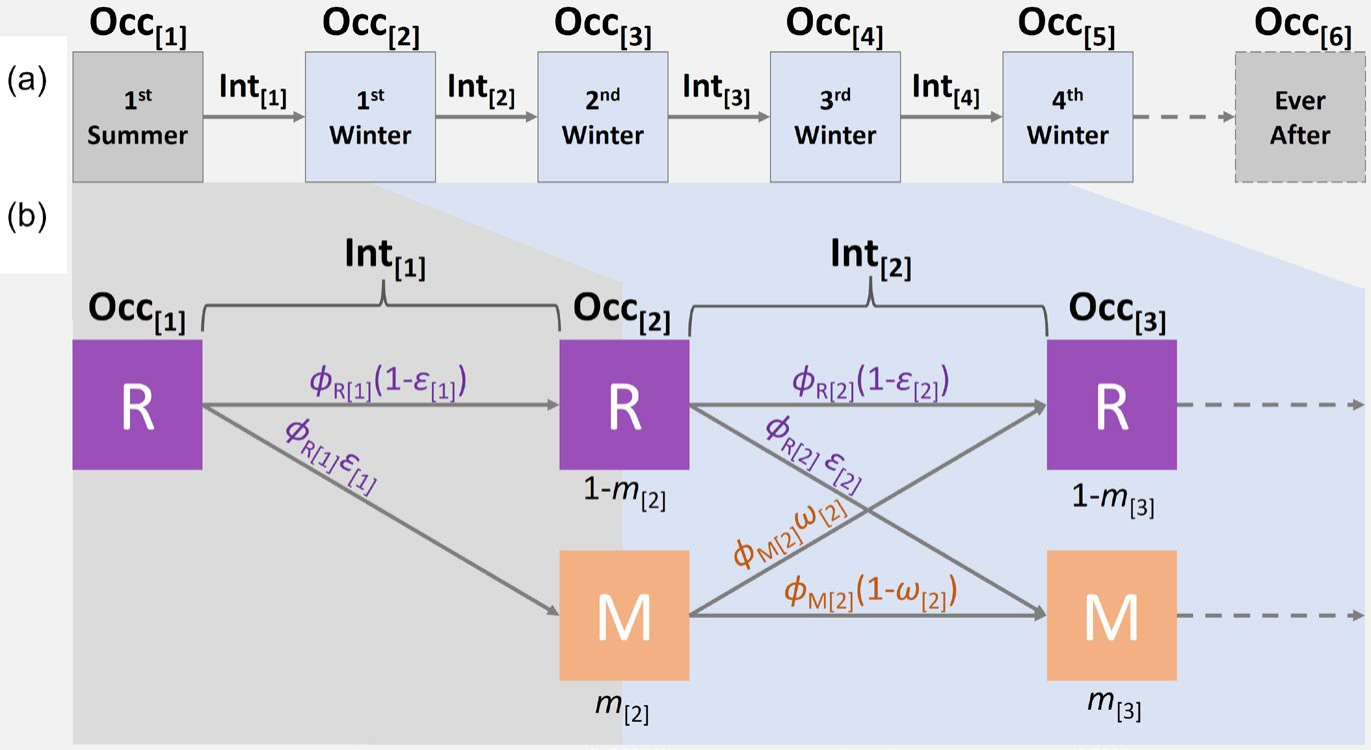
Summaries of (a) modelled occasion (Occ) and interval (Int) structure, comprising the initial fledging occasion, the four focal winter occasions (1 November–31 January each year, blue shading), and the final ‘ever after’ occasion, and (b) key aspects of model structure, showing the transition probabilities between resident (R) and migrant (M) states between occasions. All individuals are resident in occasion 1 (fledging). Parameters are depicted for the transitions between occasions 1 and 2 (grey shading) and between occasions 2 and 3 and subsequently (blue shading), and are defined in Table 1. Analogous transitions continue to occasion 5 (i.e. for the focal intervals between the first and fourth winter, full details in Supporting Information S3).

Quantifying initial expression and subsequent plasticity and selection in early‐life migration versus residence requires estimating the probabilities that individuals survive and transition between resident and migrant states between consecutive non‐breeding seasons. To achieve this while accounting for spatio‐temporal variation in field detection of colour‐ringed individuals, we devised a MS‐CMR model that simultaneously estimates survival probabilities for current residents (*ϕ*_R_) and migrants (*ϕ*_M_), movement probabilities representing departure for residents (i.e. transitioning to migrant, *ε*) and return for migrants (i.e. transitioning to resident, *ω*) and hence probabilities of not moving (remaining resident, 1 − *ε*, and remaining migrant, 1 − *ω*), alongside detection probabilities (Figure 1; Acker, Daunt, et al., 2021; Ugland et al., 2024).

The model structure comprised five primary discrete ‘occasions’ for each cohort, generating four intervening intervals during which individuals can survive (or die) and move (or remain; Figure 1). The first occasion comprised the cohort's natal breeding season, when all individuals fledged from IoM and hence were initially defined as ‘resident’. The next four occasions comprised the cohort's subsequent four winters (each defined as 1 November to 31 January; Figure 1; Supporting Information S2). These occasions represent successive ages spanning the first 3.5 years from fledging (beyond which cumulative mortality was too high for cohort‐level analyses). This formulation allows direct estimation of survival and movement probabilities representing transitions from resident to migrant between fledging and the first winter, then transitions between resident and migrant (in both directions) through the intervals between each subsequent consecutive winter (Figure 1). These primary parameters can then be combined to quantify between‐winter plasticity and selective disappearance (Section 2.4). Our current objectives concern parameters encompassing the second to fifth occasions (i.e. first to fourth winters), where our model quantifies survival and movement probabilities between consecutive non‐breeding seasons (not between consecutive breeding seasons, as done in many demographic studies).

To ensure that all parameters for the focal occasions and intervals are identifiable, we additionally modelled a sixth ‘ever after’ occasion indicating whether individuals were ever observed after the fourth winter (i.e. the fifth occasion; Figure 1; Supporting Information S2). This formulation allows estimation of detection probability on the fifth occasion, and hence survival probability from the fourth to the fifth occasion: these parameters are typically non‐identifiable for the final occasion and interval of fully time‐dependent capture‐mark‐recapture analyses. Accordingly, parameters for the sixth occasion, and the interval between the fifth and sixth occasion, are nuisance parameters that are not themselves biologically interpretable. Since long‐term ring‐recovery data imply that permanent emigration from the study area is very rare, estimated apparent survival probabilities represent can be interpreted as true survival (Supporting Information S2).

We used the resightings to create state‐specific encounter histories (i.e. occasion‐specific summaries of observation events) for all 9358 colour‐ringed individuals. Within each of the four focal winter occasions, individuals observed on IoM or at adjacent day roosts (used between foraging bouts) were classed as currently resident, while individuals observed elsewhere were classed as currently migrant (assignment details in Supporting Information S2). The defined mid‐winter occasion durations reduce the probability of incorrectly assigning migrant individuals as resident due to early or late movements to or from IoM, while encompassing multiple winter resighting surveys and hence allowing many migrants to be observed.

Our current analyses focus on the distinction between residents and migrants, not on migrant destinations. However, to handle spatio‐temporal heterogeneity in detection probability stemming from known variation in resighting survey effort, we split the migrants into two states, comprising intensively‐ and non‐intensively surveyed migrant sites (hereafter M1 and M2, respectively). Since these two migrant states were defined solely based on observer effort, we had no expectation that movement or survival probabilities would differ between them. Hence, for each cohort and interval, migrant survival and return probabilities were assumed equal across wintering locations, and therefore estimated as single parameters applying across the M1 and M2 states (Supporting Information S2). Since detection probability in M2 is by definition low, this formulation also effectively handles alive migrants that moved to any completely unsurveyed locations during the sub‐adult phase (Supporting Information S2). It requires further underlying movement parameters (explained in Supporting Information S3) that are necessary structural parts of our MS‐CMR model but not directly biologically informative for current purposes.

### 2.3 Model analyses

We coded and fitted the MS‐CMR model in a Bayesian framework using STAN, called from R v4.3.2 (R Core Team, 2021) with package rstan v2.32.5 (Stan Development Team, 2024). All movement, survival and detection parameters were occasion‐ and cohort‐specific, with single movement and survival parameters estimated across both migrant states (Supporting Information S3; Ugland et al., 2024). To achieve this with minimal complexity, models were fitted for each cohort separately: this entailed no loss of information since there were no shared parameters across cohorts. Sex effects were not modelled, as juvenile sexes are largely unknown prior to recruitment of survivors. However, the degree of partial migration, and associated selection, is not strongly sexually dimorphic in adult shags (Acker et al., 2023; Acker, Daunt, et al., 2021). We specified vague uniform priors (U [0, 1]) on all probabilities apart from detection in the M2 state. Here, we specified a Beta [0.5, 6] prior, giving greatest density between 0 and 0.25. This prior reflects the known low survey intensity and resulting low detection probability in M2, while still allowing the possibility of values between 0 and 1 (see Supporting Information S3 for explanations; Ugland et al., 2024). We ran *n* = 4 MCMC chains with 1000 warmup iterations then 4000 monitored iterations, generating 16,000 posterior samples.

MCMC diagnostics revealed highly satisfactory posterior sampling. All parameters had *R̂* ≤1.001, with effective sample sizes always >3000 (and typically >4000, therefore greatly exceeding 100*n*). Posterior predictive checks, assessing the degree to which estimated model parameters can accurately reproduce the observed data, confirmed good model fit (Supporting Information S4).

### 2.4 Quantifying plasticity and selection: Derived biological quantities

To quantify key biological effects of interest, including magnitudes of cohort‐specific partial migration, plasticity, selection and their joint action, we computed the full posterior distributions of combinations of primary parameters that were directly estimated by the MS‐CMR model (hereafter ‘derived parameters’; Table 1).

**TABLE 1:** Key parameters with equations and explanations, including (a) primary parameters that are directly estimated by the MS‐CMR model (all concerning intervals between consecutive occasions), and derived parameters that (b) quantify focal biological effects and (c) partition relative effects of plasticity and selective disappearance on the migratory fraction.

	Parameters	Equation	Explanation
(a)	$\phi_{R\left[t\right]}$		Resident survival probability for focal interval
	$\phi_{M\left[t\right]}$		Migrant survival probability for focal interval
	$\varepsilon_{\left[t\right]}$		Probability of departing from the resident state conditional on survival for focal interval
	$\omega_{\left[t\right]}$		Probability of returning from the migrant state conditional on survival for focal interval
(b)	$m_{\left[t\right]}$	$m_{\left[2\right]}=\varepsilon_{\left[1\right]}$ $m_{\left[t\right]}=\frac{\left(1-m_{\left[t-1\right]}\right)\phi_{R\left[t-1\right]}\varepsilon_{\left[t-1\right]}+m_{\left[t-1\right]}\phi_{M\left[t-1\right]}\left(1-\omega_{\left[t-1\right]}\right)}{P_{A\left[t\right]}}$	Migratory fraction (proportion of alive individuals that are migrants) for initial (*m*_[2]_) and subsequent occasions [*t* >2]
	$\Delta_{\phi \left[t\right]}$	$\phi_{M\left[t\right]}-\phi_{R\left[t\right]}$	Difference in survival probability between residents and migrants for focal interval (positive values imply selection for migration)
	$P_{A\left[t\right]}$	$\left(1-m_{\left[t\right]}\right)\phi_{R\left[t\right]}+m_{\left[t\right]}\phi_{M\left[t\right]}$	Proportion of individuals that survive through the focal interval among all individuals alive at the starting occasion
	$P_{S\left[t\right]}$	$\frac{\left(1-m_{\left[t-1\right]}\right)\phi_{R\left[t-1\right]}\left(1-\varepsilon_{\left[t-1\right]}\right)+m_{\left[t-1\right]}\phi_{M\left[t-1\right]}\left(1-\omega_{\left[t-1\right]}\right)}{P_{A\left[t-1\right]}}$	Proportion of individuals that are in the same state as they were in previous occasion, among all alive individuals
	$P_{\mathrm{RM}\left[t\right]}$	$\frac{\left(1-m_{\left[t-1\right]}\right)\phi_{R\left[t-1\right]}\varepsilon_{\left[t-1\right]}}{P_{A\left[t-1\right]}}$	Proportion of individuals that were previously residents and became migrants, among all alive individuals
	$P_{\mathrm{MR}\left[t\right]}$	$\frac{m_{\left[t-1\right]}\phi_{M\left[t-1\right]}\omega_{\left[t-1\right]}}{P_{A\left[t-1\right]}}$	Proportion of individuals that were previously migrants and became residents, among all alive individuals
	$\Delta_{m\left[t\right]}$	$m_{\left[t+1\right]}-m_{\left[t\right]}$	Difference in *m* between occasions (positive values imply increased migration)
(c)	$m_{\mathrm{sel}\left[t\right]}$	$\frac{m_{\left[t-1\right]}\phi_{M\left[t-1\right]}}{P_{A\left[t-1\right]}}$	Expected *m* with no plasticity (i.e. resulting only from selection) for focal occasion
	$\Delta_{m.\mathrm{sel}\left[t\right]}$	$m_{\mathrm{sel}\left[t+1\right]}-m_{\left[t\right]}$	Change in *m* due to selection for focal interval (positive values imply increased migration)
	$\Delta_{m.\mathrm{pl}\left[t\right]}$	$m_{\left[t+1\right]}-m_{\mathrm{sel}\left[t+1\right]}$	Change in *m* due to plasticity for focal interval (positive values imply increased migration)

*Note*: Proportions are calculated for each occasion, while differences are calculated for intervals between occasions. Subscripts denote occasions [*t*], where intervals are defined by their starting occasion (Figure 1).

First, for each cohort in each winter (and hence age), we quantified the expected proportion of surviving individuals that is currently migrant (hereafter the ‘migratory fraction’, *m*). For the first winter after fledging (occasion 2), *m* is simply the proportion of individuals that transitioned to the migrant state (the initial estimate of *ε*). For subsequent winters (occasions 3–5), *m* was calculated as a function of *ε*, *ω*, *ϕ*_R_ and *ϕ*_M_ to give the proportion of individuals that transitioned to or remained migrant out of all surviving individuals from the previous winter (Table 1; Ugland et al., 2024).

Second, for each cohort, we quantified the expected proportion of individuals that remained in the same state (i.e. migrant or resident) between consecutive winters (i.e. between occasions 2–5, termed *P*_S_; Table 1; Supporting Information S5) among all surviving individuals. Hence, *P*_S_ represents the net degree of between‐winter repeatability versus plasticity. We then partitioned the degree of between‐winter plasticity (i.e. 1 − *P*_*s*_) into the expected proportions of residents that transitioned to migrant (*P*_RM_) and migrants that transitioned to resident (*P*_MR_; Table 1). To calculate *P*_S_, *P*_MR_ and *P*_RM_ we first derived the expected proportion of individuals that survived through the focal interval out of the individuals alive at the beginning of the interval (*P*_A_; Table 1). Note that *P*_MR_ and *P*_RM_ differ from the directly estimated departure (*ε*) and return (*ω*) probabilities because the former are expected state proportions among all surviving individuals (whatever their previous or current state) while the latter are movement probabilities conditional on survival in each starting state (Table 1). In addition, to directly quantify the expected proportions of surviving individuals that remained in the same state between non‐consecutive winters (e.g. the first to fourth winters as represented by occasions 2 and 5), we calculated *P*_S_ from additional models fitted to reduced encounter histories that excluded the intervening winter occasions (Supporting Information S5).

Third, we quantified the degree to which the first departure (migration) probability of each cohort, and hence the initial *m* (Figure 1), predicted subsequent departure (*ε*) and/or return (*ω*) probabilities, and subsequent values of *m*, using standard linear regressions and correlations (Supporting Information S6). We thereby reveal how migratory plasticity reshapes the pattern of among‐cohort variation in partial migration. Here, positive relationships between initial *m* and subsequent *ε* would indicate that initially highly migratory cohorts were also more likely to migrate subsequently (i.e. reinforcing departures). Meanwhile, positive relationships between initial *m* and subsequent *ω* would indicate that initially highly migratory cohorts were subsequently more likely to revert to residence (i.e. compensatory returns). Overall, strong positive relationships between initial and subsequent *m*, would indicate that cohorts remain similarly ordered in their degree of partial migration across successive winters, whereas strong negative relationships would indicate a reversal in order where initially more migratory cohorts systematically become more resident and vice versa. Meanwhile, weak or no relationships would indicate that the degree of partial migration becomes reordered across cohorts, either randomly or by converging on a common value (Supporting Information S6).

Fourth, to quantify the direction and magnitude of survival selection on migration versus residence between winters for each cohort and age, and hence quantify variation in selective disappearance, we calculated the difference between *ϕ*_M_ and *ϕ*_R_ (*Δ*_*ϕ*_; Table 1). Here, positive and negative values indicate selection for migration and residence, respectively. Since most annual mortality occurs through mid‐late winter, and many sub‐adults remain in their winter location through initial summers, variation in survival and resulting selection will predominantly reflect starting winter state.

Fifth, to decompose overall effects and reveal to what degree between‐winter changes in *m* within cohorts were caused by plasticity, selective disappearance, or some combination of both, we first estimated the expected *m* following selection but before any movements (i.e. without plasticity; *m*_sel_; Table 1). The change in *m* attributable to selection (*Δ*_m.sel_) was calculated as the difference between *m*_sel_ and the starting *m*. The change in *m* attributable to plasticity (*Δ*_m.pl_) was then calculated as the difference between the subsequent *m* and *m*_sel_ (Table 1). In all cases, positive and negative differences, respectively, indicate increased and decreased migration between consecutive winters.

To explicitly quantify the degree to which expected changes in *m* due to within‐cohort plasticity and selective disappearance were reinforcing or counter‐acting, we calculated the proportion of posterior estimates for each cohort where *Δ*_m.pl_ and *Δ*_m.sel_ had the same sign (*P*_C_). Here, *P*_C_ values close to zero and one imply that *Δ*_m.pl_ and *Δ*_m.sel_ acted in the same or opposite directions, respectively, indicating reinforcing or counter‐acting effects on *m* (in either phenotypic direction). Finally, we tested for relationships between *Δ*_m.sel_ and *Δ*_m.pl_, such that the absolute magnitude (and hence contribution) of either one was systematically greater than the other, or that the magnitude of *Δ*_m.sel_ predicted the magnitude of *Δ*_m.pl_ through a negative regression (implying that plasticity compensated for preceding selection, details in Supporting Information S7).

### 2.5 Summary statistics

For all primary and derived parameters, full posterior distributions of the estimates were summarized as posterior means and 95% credible intervals (95% CI denoted [0.025, 0.975]). Some primary and derived parameters are expected to be estimated with considerable uncertainty, for example, for occasions and intervals following high cohort mortality, where few individuals remain in particular alive states. However, such uncertainty does not necessarily impede overall inferences or interpretations of derived effects. For derived differences (*Δ*_*ϕ*_, *Δ*_*m*_, *Δ*_m.sel_ and *Δ*_m.pl_), 95% CIs that exclude zero are indicative of strong evidence of a difference (i.e. 97.5% probability that the posterior mean is positive or negative, implying statistically meaningful inferences regardless of the effect size). Evidence for divergent or reinforcing effects of plasticity and selection on *m* is interpreted as strong if the proportions of posterior estimates that are in the same direction are >0.975 or <0.025 and moderate if ≥0.75 or ≤0.25.

We additionally summarized primary and derived parameters across all 11 cohorts by computing and summarizing full posterior distributions of cross‐cohort means and among‐cohort variances. These summary statistics reveal overall patterns of age‐specific changes in means, and among‐cohort variation, in partial migration, migratory plasticity and selective disappearance. Extreme winter storms led to very high first‐winter mortality for the 2012 and 2013 cohorts (Ugland et al., 2024). Estimates of subsequent movement and survival parameters for these two cohorts were consequently particularly imprecise. Accordingly, cross‐cohort means and among‐cohort variances were computed with and without these two cohorts. These exclusions did not alter conclusions except where noted (all results in Supporting Information S8 and S9). Another extreme winter mortality event occurred in 2023, causing very low survival for the 2020 cohort from their third to fourth winter. Since this represents the final timepoint of our current analyses, no subsequent parameters were affected.

Full annotated code, data and numerical results for all parameters are archived in the Dryad repository (Ugland et al., 2026).

## 3 RESULTS

### 3.1 Among‐cohort variation in early‐life partial migration

**FIGURE 2:**
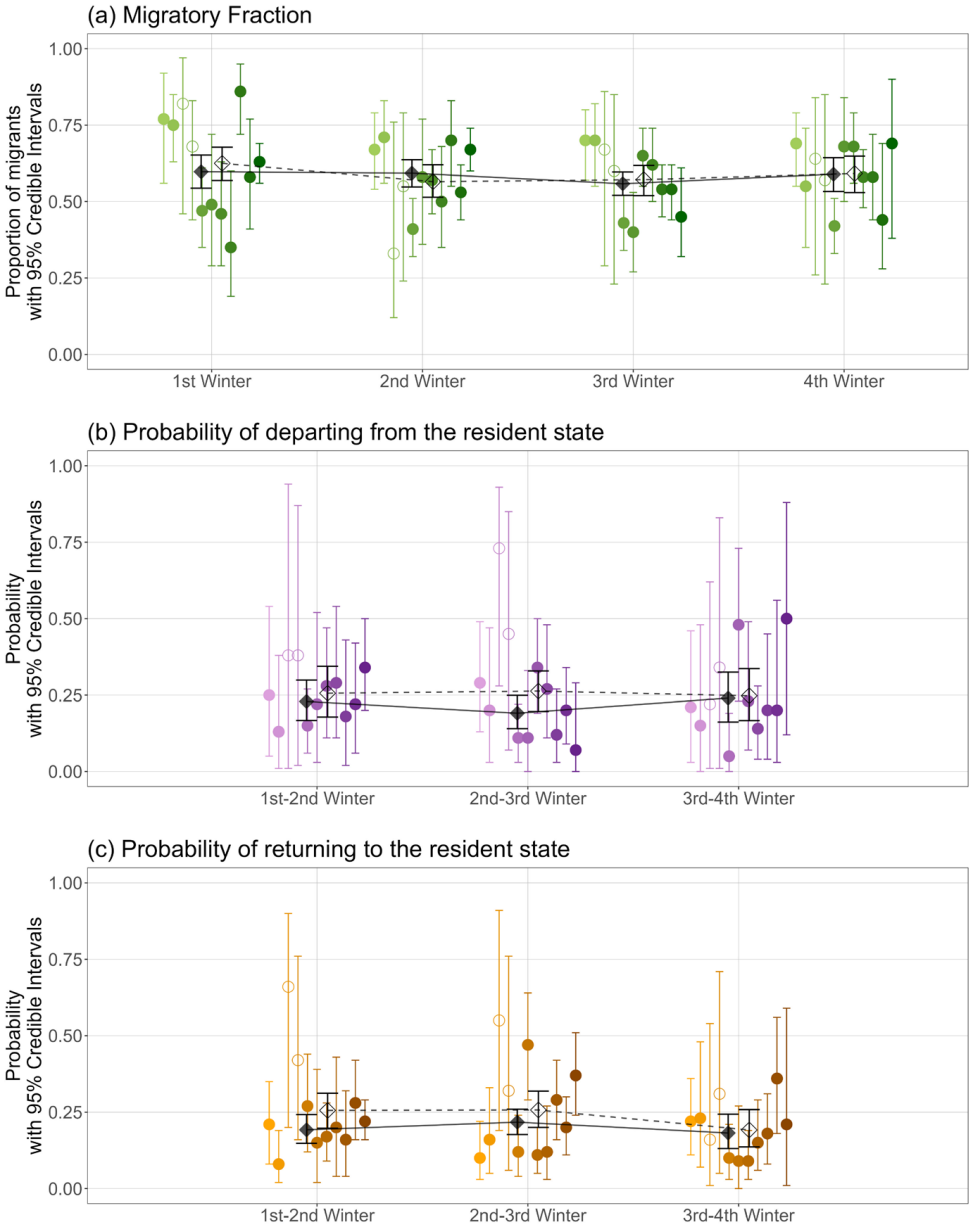
Cohort‐specific estimates of (a) occasion‐specific migratory fraction *m* (Table 1), and interval‐specific probabilities of (b) departing *ε* from and (c) returning *ω* to the resident state. Coloured points and lines show the posterior mean for each cohort (2010–2020, light–dark) with 95% credible intervals (95% CI). Black diamonds and lines show the cross‐cohort posterior means and associated 95% CIs including (open diamonds) and excluding 2012–2013 (filled diamonds).

In the first winter following fledging the mean migratory fraction (*m*) across all 11 cohorts, quantifying the overall degree of partial migration, was 0.63 [95% CI 0.57, 0.68] (Figure 2a). However, there was notable among‐cohort variation: posterior mean *m* varied from 0.35 [0.19, 0.60] in 2017 to 0.86 [0.72, 0.95] in 2018 (overall among‐cohort variance 0.04 [0.02, 0.05]). Cohorts therefore ranged from initially substantially resident to initially highly migrant (Figure 2a).

The cross‐cohort mean *m* remained very similar across the three subsequent winters (0.57 [0.51, 0.62], 0.57 [0.52, 0.62], and 0.59 [0.53, 0.65] for the second, third and fourth winters, respectively; Figure 2a). Hence, the overall degree of partial migration did not change across these ages on average. However, the among‐cohort variance halved after the first winter, then did not decrease further subsequently (0.02 [0.01, 0.04], 0.02 [0.01, 0.03], and 0.02 [0.01, 0.04] for the second to fourth winters, respectively). This shows that *m* slightly converged across cohorts after the first winter, although notable among‐cohort variation remained (Figure 2a).

### 3.2 Repeatability and plasticity

**FIGURE 3:**
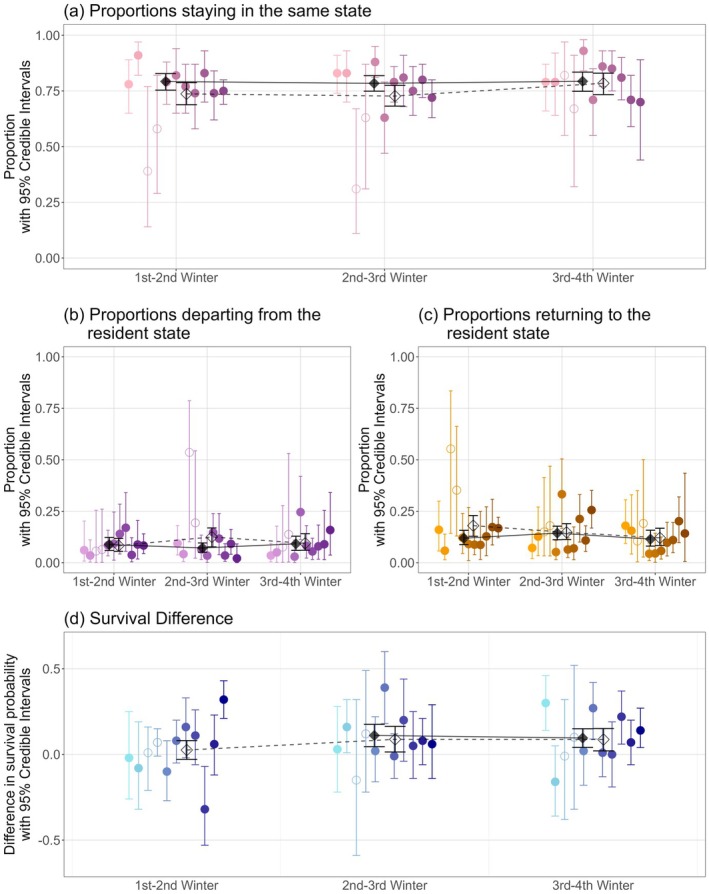
Estimated proportions of surviving individuals from each cohort that (a) remain in the same state across consecutive occasions, or (b) depart from or (c) return to the resident state. (d) Estimated difference in survival probability between residents and migrants. Coloured points and lines show the posterior mean for each cohort (2010–2020, light–dark) with 95% credible intervals (95% CI). Black diamonds and lines show the cross‐cohort posterior means and associated 95% CIs including (open diamonds) and excluding 2012–2013 (filled diamonds). On (d), points above and below zero indicate selection for migration and for residence, respectively.

The overall approximate stasis of the cross‐cohort mean *m* across successive winters (Figure 2a) partly reflected high underlying individual repeatability of migration versus residence. The expected proportions of surviving individuals that remained in the same state between consecutive winters (i.e. remained resident or migrant across consecutive ages) were high and stable (*P*_S_ of 0.74 [0.69, 0.79], 0.73 [0.68, 0.77] and 0.78 [0.73, 0.83] between the first‐second, second‐third and third‐fourth winters, respectively, i.e. approximately 75%). This outcome was broadly similar for all cohorts (excluding 2012 and 2013 where substantial mortality had already occurred; Figure 3a; Supporting Information S8). Additional models showed that repeatability was also high between non‐consecutive winters (*P*_S_ of 0.68 [0.62, 0.74], 0.65 [0.58, 0.72] and 0.73 [0.68, 0.79] between the first‐third, first‐fourth and second‐fourth winters, respectively; Supporting Information S5).

However, there was also considerable within‐cohort phenotypic plasticity manifested as individual switches between resident and migrant states between consecutive winters. Here, the cross‐cohort mean probabilities of moving from resident to migrant (*ε*) between the first‐second, second‐third and third‐fourth winters were 0.26 [0.18, 0.34], 0.26 [0.20, 0.33] and 0.25 [0.17, 0.34], respectively, and the cross‐cohort mean probabilities of moving from migrant to resident (*ω*) were similar (0.26 [0.20, 0.31], 0.26 [0.20, 0.32] and 0.19 [0.14, 0.26], respectively; Figure 2b,c). Accordingly, conditional on starting state and state‐dependent survival, plastic movements between consecutive winters on average occurred with similar probabilities in both directions.

Weighting by the proportions of individuals in each starting state (Table 1) showed that, of the ca. 25% of surviving individuals that changed phenotype between consecutive winters (i.e. were not phenotypically repeatable), on average slightly higher proportions of migrants became residents than residents became migrants (cross‐cohort means of *P*_MR_ = 0.18 [0.13, 0.23], 0.15 [0.11, 0.19], 0.12 [0.08, 0.17] and *P*_RM_ = 0.08 [0.06, 0.12], 0.13 [0.08, 0.17], 0.09 [0.06, 0.14] for the first‐second, second‐third and third‐fourth winters, respectively; Figure 3b,c). This tendency for plastic shifts towards residence between the first and second winters was greater when excluding the 2012 and 2013 cohorts (Supporting Information S8). These outcomes reflect initial *m* >0.5, generating differing values of *P*_MR_ and *P*_RM_ despite similar values of *ε* and *ω*.

### 3.3 Forms of plasticity

Individual cohorts did not show consistent *ε* or *ω* values across winters (Figure 2; Supporting Information S8). Specifically, among‐cohort variation in subsequent *ε* and *ω* was not strongly associated with among‐cohort variation in initial *m*. The regression slopes and correlations of *ε* and *ω* on initial *m* were weak (all regression slopes and correlation coefficients <0.27 with 95% CIs overlapping zero; Supporting Information S6). There was therefore no evidence that plasticity systematically reinforced or compensated for initial among‐cohort variation in migration. Further, while first‐winter *m* was positively associated with second‐winter *m* when the 2012 and 2013 cohorts were excluded (regression slope 0.45 [0.15, 0.74], correlation coefficient 0.68 [0.25, 0.93]), the association was weaker across all cohorts (slope 0.21 [−0.18, 0.64], correlation 0.36 [−0.06, 0.80]), and first‐winter *m* was not associated with third‐ or fourth‐winter *m* (slopes 0.19 [−0.15, 0.50], 0.07 [−0.27, 0.40] and correlations 0.28 [−0.18, 0.64], 0.10 [−0.35, 0.55], respectively; Supporting Information S6). Hence, early‐life plasticity, in conjunction with any selection, re‐ordered the initial pattern of among‐cohort variation in partial migration.

### 3.4 Selective disappearance

Across all 11 cohorts, the cross‐cohort mean survival probability from fledging to the first winter was 0.74 [0.69, 0.78]. Then, between the first and second winters, the cross‐cohort mean survival probabilities for residents and migrants (conditional on starting state) were *ϕ*_R_ = 0.49 [0.45, 0.54] and *ϕ*_M_ = 0.52 [0.49, 0.55]. There was consequently no evidence of substantial selective disappearance through the first‐second winter on average (*Δ*_*ϕ*_ = 0.03 [−0.03, 0.08]; Figure 3d). Subsequently, cross‐cohort mean survival probabilities for residents and migrants increased to *ϕ*_R_ = 0.67 [0.61, 0.72] and *ϕ*_M_ = 0.76 [0.71, 0.81], and *ϕ*_R_ = 0.68 [0.63, 0.72] and *ϕ*_M_ = 0.76 [0.71, 0.81] for the second‐third and the third‐fourth winters, respectively, giving *Δ*_*ϕ*_ = 0.09 [0.01, 0.16] and 0.09 [0.02, 0.15], respectively (Figure 3d; Supporting Information S9). Hence, there was strong overall evidence of survival selection, where migrants had higher survival probabilities than residents on average across cohorts, implying selective disappearance of residents through the second‐fourth winters following fledging.

However, there was also notable among‐cohort variation in the magnitude and direction of survival selection (Figure 3d; and in the underlying migrant and resident survival probabilities *ϕ*_M_ and *ϕ*_R_; Supporting Information S9). Specifically, there was evidence of selection for migrants (i.e. selective disappearance of residents) in seven instances, including very substantial differences in survival probability (*Δ*_*ϕ*_: first‐second winters in 2020: 0.32 [0.21, 0.43]; second‐third winters in 2011: 0.16 [0.01, 0.32] and 2015: 0.39 [0.18, 0.60]; third‐fourth winters in 2010: 0.30 [0.14, 0.46], 2015: 0.27 [0.09, 0.42], 2018: 0.22 [0.06, 0.37] and 2020: 0.14 [0.04, 0.27]). Meanwhile, there was one instance of selection for residents (i.e. selective disappearance of migrants, first‐second winter in 2018: *Δ*_*ϕ*_ = −0.32 [−0.53, −0.07]) and little or no evidence for selection in other cohort‐years.

### 3.5 Joint impacts of plasticity and selective disappearance

**FIGURE 4:**
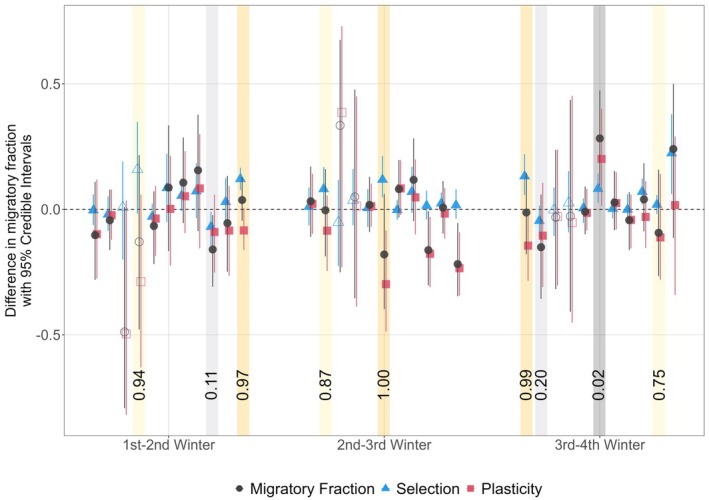
Estimated expected differences in migratory fraction *m* between occasions with only selection (*Δ*_m.sel_; blue triangles) or only plasticity (*Δ*_m.pl_; pink squares) compared to the actual difference in *m* (*Δ*_*m*_; grey circles) for each cohort (2010–2020 left to right) and interval with 95% credible intervals (95% CI). Points above and below zero, respectively, indicate increased and decreased migration between occasions. Coloured shading indicates the proportions of posterior estimates of *Δ*_m.sel_ and *Δ*_m.pl_ that converge (grey) or diverge (yellow; *P*_c_). Numbers are the proportions of posterior estimates that diverge, with strong (>0.975, <0.025) and weak (≥0.75, ≤0.25) evidence indicated by darker and lighter shading, respectively.

Within cohorts and intervals, there were clear instances of both reinforcing and counter‐acting effects of plasticity and selective disappearance on *m*, leading to both increases and decreases in *m* between consecutive winters, with no clearly consistent outcomes (Figure 4). For example, there was strong evidence of reinforcing plasticity and selection, where both *Δ*_m.sel_ and *Δ*_m.pl_ acted in the same direction and hence amplified the net change in *m*, for the third‐fourth winter for the 2015 cohort (*P*_C_ = 0.02) and moderate evidence for the first‐second winter for the 2018 cohort (*P*_C_ = 0.11) and third‐fourth winter for the 2011 cohort (*P*_C_ = 0.20; Figure 4). However, the direction of effects differed, leading to increased *m* in the 2015 cohort and decreased *m* in the 2018 and 2011 cohorts (Figure 4).

Conversely, there were also examples of strongly counter‐acting effects of plasticity and selection, where *Δ*_m.sel_ and *Δ*_m.pl_ acted in opposite directions and consequently reduced the net change in *m*. This was most striking for the first‐second winter for the 2020 cohort (*P*_C_ = 0.97), the second‐third winter for the 2015 cohort (*P*_C_ = 1.00) and the third‐fourth winter for the 2010 cohort (*P*_C_ = 0.99; Figure 4). Moderate evidence for counter‐acting effects (*P*_C_ ≥0.75) also occurred in three other intervals (Figure 4). In these instances, selection always increased migration while plasticity always increased residence. Overall, *Δ*_m.sel_ did not consistently exceed *Δ*_m.pl_ (or vice versa) and *Δ*_m.sel_ did not strongly predict *Δ*_m.pl_ across cohorts and ages (Figure 4; Supporting Information S7).

## 4 DISCUSSION

Changes in mean life‐history trait values across early life, and resulting impacts on population demography and dynamics, fundamentally result from joint actions of labile plasticity and selective disappearance following initial development (Forsythe et al., 2024; van de Pol et al., 2012; van de Pol & Verhulst, 2006). However, the degrees to which these processes vary among cohorts, and the resulting dynamic cohort effects, are rarely quantified for any trait. We show that, in European shags, the key life‐history trait of facultative seasonal migration versus residence (and the resulting degree of partial migration) is on average consistently expressed through the first four winters following fledging, with little age‐specific change across cohorts. However, this overall cross‐cohort stasis hides considerable within‐ and among‐cohort variation in initial and subsequent migration across ages, and in underlying plasticity and selective disappearance. These processes had strongly reinforcing or counter‐acting effects within different cohorts, acting to increase or decrease between‐year changes in cohort‐specific degrees of partial migration in both directions. Apparent overall early‐life phenotypic stability was therefore not fixed, but was underpinned by considerable dynamism in initial expression, plasticity and selection, and hence in the pattern of among‐cohort variation. This implies that mechanistic within‐cohort analyses will be required to predict how future age‐specific phenotypic changes could rapidly emerge given changes in spatio‐seasonal environmental conditions that systematically alter patterns of plasticity and selection.

### 4.1 Early‐life cohort effects on partial migration

In shags, the cross‐cohort mean degree of partial migration was similar across the first four winters following fledging, with approximately 60% migrants at each age on average. Similarly, approximately 58% of breeding adult shags were migrants during 2010–2018 (Acker et al., 2023). There was consequently no discernible average decrease in migration from sub‐adult to full adulthood. This contrasts with some other partially migratory populations, where residence increases through early life, even such that migration is largely eliminated following recruitment (e.g. North Atlantic right whales *Eubalaena glacialis*: Gowan et al., 2019; red kites *Milvus milvus*: Chan et al., 2024; Witczak et al., 2024; Eurasian sparrowhawks *Accipter nisus* and common buzzards *Buteo buteo*: Holte et al., 2017; but see Eggeman et al., 2016). However, such age‐specific changes are typically quantified by pooling cohorts into broader age‐classes, potentially obscuring within‐ and among‐cohort variation and dynamics.

Indeed, considerable among‐cohort variation in the degree of partial migration was evident in shags, particularly in the first winter following fledging (Figure 2). Since additive genetic variance underlying early‐life migration is small relative to environmental variance (heritability 0.1–0.2; Fortuna et al., 2024), and consecutive cohorts are produced by greatly overlapping sets of parents (median parent age 6 years, inter‐quartile range 4–10, range 2–22), initial among‐cohort variation must substantially reflect cohort‐specific pre‐ or post‐fledging environmental effects rather than micro‐evolutionary change. Yet, initial cohort‐specific values of *m* did not strongly predict subsequent values, showing that the relative degrees of partial migration exhibited by each cohort changed across successive winters (and hence ages). This implies that initial environmental effects are partially overridden by subsequent effects, through plasticity and/or selection.

Among‐cohort variation in early‐life winter location was previously highlighted in greater flamingos (*Phoenicopterus roseus*), where proportional use of one resident and three migrant first‐winter locations varied considerably among 37 cohorts (Sanz‐Aguilar et al., 2012). However, cohort‐specific site fidelity across subsequent winters was not estimated. Otherwise, despite increasing interest in early‐life movements, the form and magnitude of among‐cohort variation is rarely quantified. This is likely because remote tracking technologies are still typically challenging to apply to numerous juveniles (Hazen et al., 2012; Shillinger et al., 2012). Consequently, data are commonly pooled across cohorts (e.g. Chan et al., 2024; Sergio et al., 2014; Witczak et al., 2024). Our MS‐CMR analyses of bulk colour‐ring resighting data reveal clear evidence of substantial and dynamic among‐cohort variation in early‐life partial migration across successive winters (despite inevitable imprecision in some estimates).

### 4.2 Early‐life plasticity versus repeatability

On average across cohorts, 75% of shags retained the same phenotype (either resident or migrant) between consecutive winters, representing substantial early‐life individual phenotypic repeatability (that was also evident across non‐consecutive winters; Supporting Information S5). Early‐life repeatability of seasonal migration versus residence, and resulting site fidelity, have rarely been estimated in other taxa. However, two previous studies, both on partially migratory birds, showed that >78% of pied avocets (*Recurvirostra avosetta*: Chambon et al., 2019) and >72% of greater flamingos (Sanz‐Aguilar et al., 2012) retained the same winter locations between their first and second winters. More widely, high adult repeatability in migration occurs in diverse systems, including noctule bats (*Nyctalus noctule*: Lehnert et al., 2018), mule deer (*Odocoileus hemionus*: Sawyer et al., 2019) and roach (*Rutilus rutilus*: Brodersen et al., 2014). Adult shags also show high, yet phenotype‐dependent, repeatability: >72% of migrants and >86% of residents express the same phenotype across consecutive winters (Acker et al., 2023). Together, such studies indicate that among‐individual phenotypic variation in migration versus residence may commonly be highly canalized, and could become so from young ages.

Yet, 75% early‐life repeatability leaves 25% of surviving shags that switched between residence and migration between consecutive winters, providing potential for among‐cohort variation in the degree of within‐cohort change in partial migration. Here, cross‐cohort mean expected proportions of surviving individuals switching from migrant to resident were slightly higher than from resident to migrant. Consequently, there was some evidence for directional plastic shifts towards residence across ages. However, the form and degree of plasticity was not constant across winters, and hence across ages, and partially reordered the pattern of cohort‐specific partial migration through the sub‐adult period. Such among‐cohort variation in migratory plasticity has not previously been quantified in any system and demonstrates the degree to which varying subsequent environments can gradually over‐ride effects of early‐life conditions.

### 4.3 Selective disappearance

Our analyses showed clear among‐cohort variation in the direction of survival selection on seasonal migration versus residence occurring through the first‐second winter: different cohorts experienced selection for or against migration, with no strong directionality on average (Figure 3d). ‘Fluctuating’ early‐life selection (i.e. with sign changes) occurring during the first weeks and months following fledging was previously highlighted in shags, stemming from short episodes of strong mid‐winter selection and/or cumulative effects of consistent weak selection (Ugland et al., 2024). Our current analyses reveal that some fluctuating selection also occurs through to the second winter following fledging, implying temporally varying selective disappearance. Such evidence of fluctuating survival selection in nature is rare for any trait, particularly in early life (de Villemereuil et al., 2020; Morrissey & Hadfield, 2012; Ugland et al., 2024).

Our analyses then reveal relatively consistent survival selection favouring migration through the second‐third and third‐fourth winters (Figure 3d). There was consequently some systematic selective disappearance of residents through these ages. This adds to conflicting wider evidence on the directionality of early‐life selection on migration. For example, selection for migration also occurred in European blackbirds (*Turdus merula*), where juvenile (and adult) migrants had higher survival probabilities than residents (Zúñiga et al., 2017), and in moose (*Alces alces*) where mother and hence calf survival was higher in migrants (White et al., 2014). Conversely, in immature Eurasian spoonbills (*Platalea leucorodia leucorodia*), residents and short‐distance migrants had higher survival probabilities than long‐distance migrants between winters and consecutive breeding seasons (Ferreira et al., 2024; Lok et al., 2011), and long‐distance migrants also had lower survival probabilities in juvenile greater flamingos (Sanz‐Aguilar et al., 2012). The relative fitness consequences of early‐life migration therefore vary among systems, and may scale with distance travelled.

The average survival selection against residence observed in sub‐adult shags could contribute to maintaining partial migration in this system. This is because resident adult shags typically have higher annual reproductive success than migrants, and hence higher overall annual fitness, in years without severe winter storms that cause high resident mortality (Acker, Burthe, et al., 2021). These outcomes could be counteracted by the typically higher sub‐adult migrant survival probabilities, given some degree of consistent phenotypic expression into adulthood. An analogous, but opposite, mechanism may operate in flamingos, where higher resident survival in sub‐adults preceded higher migrant survival in adults (Sanz‐Aguilar et al., 2012).

### 4.4 Joint dynamics of plasticity and selective disappearance

The average selective disappearance of residents following the second winter, combined with some directional plasticity towards residence, partially explains the overall average stasis in the degree of partial migration across ages. However, the cohort‐specific dynamics of *m* stem from the joint dynamics of plasticity and selection within cohorts. Here, our analyses revealed instances of reinforcing and counter‐acting effects, where plasticity and selection acted in the same or opposite directions, alongside instances where only one process primarily acted (Figure 4). Consequently, across cohorts, there was no evidence that plasticity and selection were consistently aligned with initial *m*, or with each other. Hence, plasticity was not clearly adaptive, where individuals switch to phenotypes that are selected for. Neither is there any clear indication of strongly compensatory spatial effects, where individuals move into space that has just been released by recent selection. Our study therefore highlights that understanding phenotypic changes through early life requires quantifying the dynamic contributions of both plasticity and selection, as quantifying either alone would fail to explain or predict observed outcomes in terms of both within‐cohort change and overall stasis (van de Pol & Verhulst, 2006).

Few previous studies have simultaneously quantified both plasticity and selection in migration. In adult shags, changing partial migration was similarly shaped by varying contributions of plasticity and selection in different years (Acker et al., 2023). Meanwhile, both processes jointly advanced departure date in pooled age‐classes of black kites (*Milvus migrans*) through early life (Sergio et al., 2014). Beyond migration, variable contributions of early‐life plasticity and selection have been found to affect other movement and behavioural traits. Plasticity, rather than selective disappearance, shaped early‐life decreases in individual repeatability of aggression in North American red squirrels (*Tamiasciurus hudsonicus*), despite among‐cohort variation in selection (Martinig et al., 2021). Similarly, plasticity, rather than selective disappearance of more mobile individuals, increased site fidelity with age in griffon vultures (*Gyps fulvus*: Acácio et al., 2024). However, few such studies on any traits or ages consider that the forms and magnitudes of plasticity and selective disappearance, and hence their joint impacts on phenotypic change, are not necessarily fixed, but can vary substantially among years and hence cohorts. Accordingly, there is considerable scope to overlook substantial within‐cohort dynamics that could generate dynamic age‐structure.

## 5 CONCLUSIONS AND IMPLICATIONS

Our study shows that both initial phenotypic expression of a key life‐history trait that directly shapes spatio‐seasonal population dynamics (i.e. seasonal migration versus residence), and also subsequent forms of labile plasticity and selective disappearance and their joint effects, can vary substantially among cohorts. Apparent cross‐age phenotypic stasis is therefore underpinned by variable underlying processes, reshaping the pattern of among‐cohort variation across ages. This implies that future directional environmental changes that affect initial expression of migration and/or affect plasticity or selection, could substantially alter the population's phenotypic composition and resulting spatio‐seasonal distribution, and rapidly reshape the overall pattern of age‐specific life‐history change. Future analyses that identify key environmental drivers of labile plasticity and selective disappearance will therefore be required to predict the dynamics of among‐cohort variation, and resulting variation in recruitment and population structure, given any scenario of environmental change. Such drivers could include spatial variation in habitat quality and prey abundance, direct or lagged effects of local extreme climatic events (Supporting Information S9), and variation in population density at the resident and migrant locations. Any such density‐dependence in migration, and in resulting survival, could further feed back to cause frequency‐dependent selection on migration that acts within or across cohorts (e.g. Chapman et al., 2011; Descamps et al., 2008; Liu et al., 2025; Taylor & Norris, 2007). However, further years of data will be required to provide sufficient power to identify combinations of covariates that explain variation in movement and survival in our system. Such work will also require further analytical developments to quantify changes in local population density that could both cause and result from movement and survival. Additionally, future analyses could quantify the degree to which expression of between‐winter plasticity in migration versus residence, and in migrant destinations, itself experiences temporally varying selection, potentially revealing additional forms of environmentally induced non‐random disappearance.

Previous studies have shown that among‐cohort variation in early‐life survival can substantially shape population age‐structure (Cooper & Kruuk, 2018; Douhard et al., 2014; Payo‐Payo et al., 2023). By focussing on the critical trait of seasonal migration versus residence, our study advances such general principles by showing how early‐life cohort effects could also generate seasonal spatial structuring. Different cohorts may consequently be differentially affected by non‐breeding season environmental conditions, potentially inducing complex spatio‐seasonal dynamics of population age‐structure given spatio‐seasonal environmental change. Cohort‐level analyses will therefore be required to yield appropriate predictions.

## AUTHOR CONTRIBUTIONS

Overarching objectives were conceived by Jane M. Reid, Francis Daunt, Paul Acker and Cassandra R. Ugland. Data extraction and analyses were conceived and implemented by Cassandra R. Ugland, Jane M. Reid, Paul Acker and Rita Fortuna. Long‐term data were collected and collated by Mark A. Newell, Sarah J. Burthe, Francis Daunt, Sarah Wanless, Michael P. Harris, Carrie Gunn, Timothy I. Morley, Robert L. Swann, Josie H. Hewitt, Erin A. Taylor and Jane M. Reid. The manuscript was drafted by Cassandra R. Ugland with input from Jane M. Reid. All authors contributed to conceptual development and manuscript editing.

## FUNDING INFORMATION

Funding for this study was provided by the UK Natural Environment Research Council (awards NE/M005186/1, NE/R000859/1 and NE/R016429/1 through the UK‐SCaPE programme delivering National Capability and award NE/Y000684/1), and the Norwegian Research Council (grants 223257 and 313570), NTNU and University of Aberdeen.

## CONFLICT OF INTEREST STATEMENT

The authors declare no conflict of interest.

## ETHICS STATEMENT

Bird ringing was licensed by the British Trust for Ornithology (permit numbers A400 and A4607).

## Supporting information


**Supporting Information S1.** Concepts of joint effects of plasticity and selective disappearance.
**Supporting Information S2.** Details of the field system, occasions and encounter histories.
**Supporting Information S3.** Details of full model structure and parameters.
**Supporting Information S4.** Details of model checks.
**Supporting Information S5.** Non‐consecutive winter repeatability.
**Supporting Information S6.** Relationships between initial *m* and subsequent movement parameters.
**Supporting Information S7.** Joint effects of plasticity and selection on partial migration.
**Supporting Information S8.** Cross‐cohort movement results.
**Supporting Information S9.** Cross‐cohort survival results.

## Data Availability

Data are available from the Dryad Digital Repository: https://doi.org/10.5061/dryad.1c59zw481 (Ugland et al., 2026).
