# Supplementary material for: Early‐life stasis in partial seasonal migration is underpinned by among‐cohort variation in migratory plasticity and selective disappearance
Source: J Anim Ecol. 2026 Jan 28;95(3):538–52. doi: 10.1111/1365-2656.70215 (PMC12957729; doi:10.1111/1365-2656.70215)
Supplement: Supplementary file 1 — Supporting Information S1. Concepts of joint effects of plasticity and selective disappearance. Supporting Information S2. Details of the field system, occasions and encounter histories. Supporting Information S3. Details of full model structure and parameters. Supporting Information S4. Details of model checks. Supporting Information S5. Non‐consecutive winter repeatability. Supporting Information S6. Relationships between initial m and subsequent movement parameters. Supporting Information S7. Joint effects of plasticity and selection on partial migration. Supporting Information S8. Cross‐cohort movement results. Supporting Information S9. Cross‐cohort survival results. [file JANE-95-538-s001.docx]

Supporting Information for: Early-life stasis in partial seasonal migration is underpinned by among-cohort variation in migratory plasticity and selective disappearance

Table of Contents

S1. Concepts of joint effects of plasticity and selective disappearance 1

S2. Details of the field system, occasions, and encounter histories 3

S3. Details of full model structure and parameters 8

S4. Details of model checks 12

S5. Non-consecutive winter repeatability 15

S6. Relationships between initial *m* and subsequent movement parameters 17

S7. Joint effects of plasticity and selection on partial migration 21

S8. Cross-cohort movement results 24

S9. Cross-cohort survival results 26

References 29

S1. Concepts of joint effects of plasticity and selective disappearance

The two processes that could alter cohort mean phenotypes across timepoints are labile phenotypic plastic (defined as phenotypic changes within individuals) and selective disappearance (defined as non-random mortality of individuals with particular phenotypes). Figure S1 illustrates potential joint impacts of the forms and magnitudes of labile plasticity and selective disappearance on a mean phenotype across timepoints. Instances where the mean phenotype does not change over time could imply that there is no plasticity and no selection (Figure S1a) or could imply that there are equal and opposite effects of both (Figure S1b), which could vary in direction. Meanwhile, if one process is not acting, phenotypic change will be completely predicted and described by the form and magnitude of the other (Figure S1c). Further, if both plasticity and selection are acting, but differ in magnitude and/or direction, the total change to the mean phenotype will either be reinforcing and hence amplified (Figure S1d) or counter-acting and hence diminished (Figure S1e) compared with the change resulting from either process alone. These processes could vary across consecutive timepoints, as could be envisaged as different sequences of Figure S1 panels a-e, generating complex sequences of phenotypic change due to the joint dynamics of labile plasticity and selective disappearance.


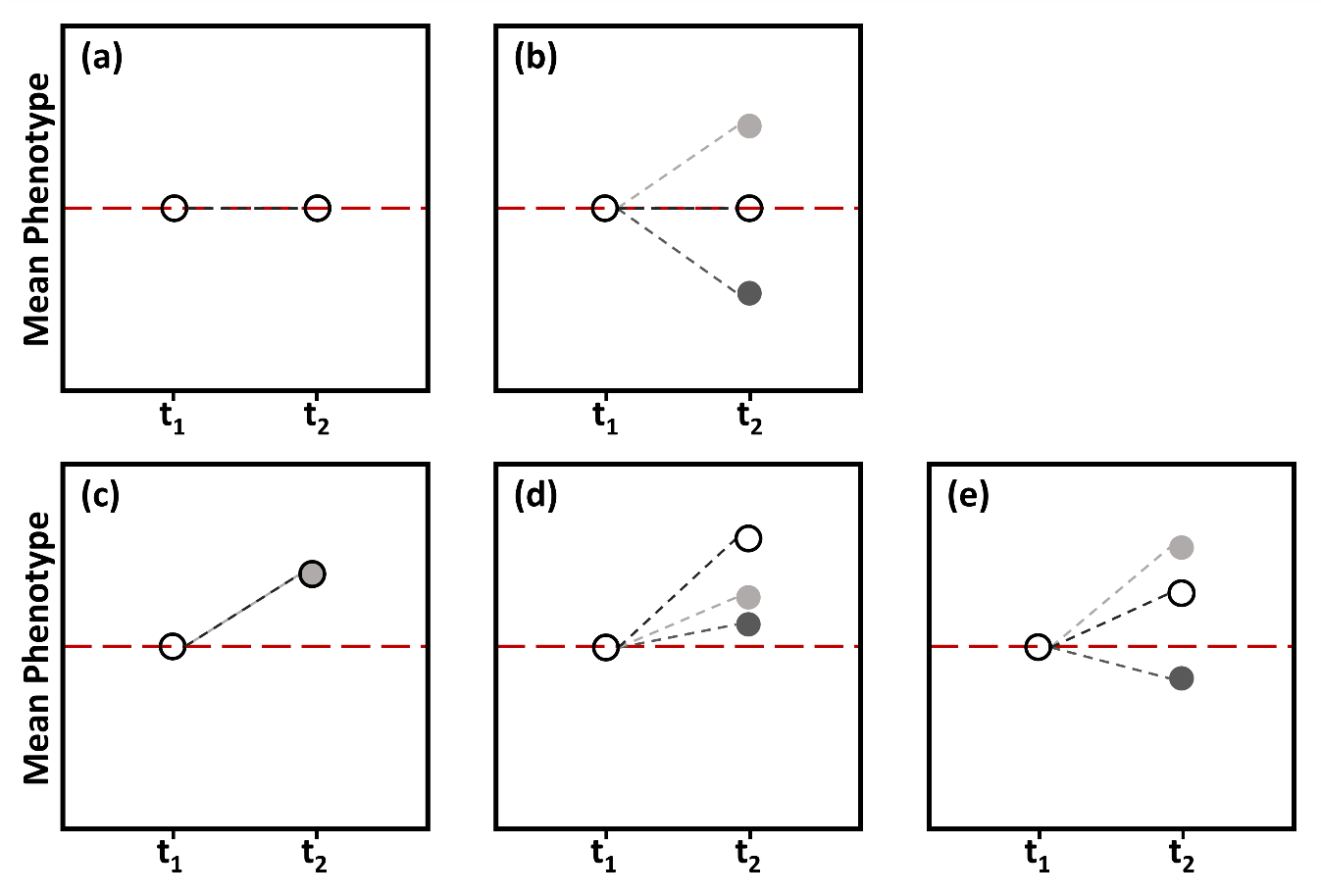
**Figure S1** Change in mean phenotype (black open points and black dashed lines) between two timepoints (t_1_ and t_2_) due to differing contributions of labile plasticity and selective disappearance (which could be either of the grey points and grey dashed lines). Scenarios comprise (a) zero plasticity and zero selection and hence zero change in mean phenotype, (b) equal and opposite effects of plasticity and selection (i.e., cancelling out), and hence zero net change in mean phenotype, (c) only plasticity or only selection, and hence change in mean phenotype that matches the effect of the one acting process, (d) plasticity and selection act in the same direction, hence the change in mean phenotype exceeds both individual processes (reinforcing effects), and (e) plasticity and selection act in opposite directions but are unequal, hence the change in mean phenotype is intermediate between the two effects (counter-acting effects, in numerous possible configurations). The dashed red line demarcates where the joint effects of plasticity and selection on mean phenotype are positive (above the line) or negative (below the line).

S2. Details of the field system, occasions, and encounter histories

Here we provide additional details of the study system and resighting data collection (as presented in Acker, Burthe et al. 2021; Acker, Daunt et al. 2021; Grist et al. 2014; Ugland et al. 2024), the structure of the occasions used for our multi-state capture-mark-recapture model, and the creation of corresponding encounter histories.

European shags are moderately long-lived seabirds (in our dataset median parent age 6 years, inter-quartile range 4-10, maximum 22) with a protracted sub-adult phase (typically lasting 3-4 winters from fledging to recruitment Aebischer, Potts, & Coulson (1995), though age at first breeding can range between 2-6 years old) during which individuals can seasonally migrate or remain resident. Throughout the focal breeding seasons (typically April through early August, with most individuals fledging during late June and July) during our study period an intensive ringing and field resighting program was undertaken on the Isle of May (hereafter ‘IoM’) by the UK Centre for Ecology and Hydrology, supported by Isle of May Bird Observatory and NatureScot. During each breeding season all nests were monitored, ringed breeding adults were identified, and chicks were individually colour-ringed at their nests before fledging (under permits A400 and A4670, licenced by the British Trust for Ornithology), and hence were subsequently field identifiable at ≤150m without need for recapture. Field resightings of non-breeding sub-adults were also recorded during the breeding season.

In the non-breeding season intensive resighting surveys focussed on known winter roosting sites along the eastern Scottish coast (i.e., IoM, Fife, Lothian, Peterhead, Fraserburgh, Portknockie, and North Sutor; Figure S2). Surveys were undertaken at least fortnightly at each key site, during which observers note the identities of all present ringed shags, as well as the time, location, and confidence of each observation. Further observations, including contributions from citizen scientists, at other locations provide low-intensity resighting data for a wider geographical area spanning the UK and Europe (Figure S2).

Resighting data were checked, validated and curated by data managers from the UK Centre for Ecology & Hydrology and University of Aberdeen. All observations were checked against ringing data and subsequent resightings, ensuring possible errors were flagged and double-checked before entering the main database. Data used in this study were extracted from the main database with filters to extract lifetime observations of individuals that were colour-ringed prior to fledging on IoM during the 2010-2020 breeding seasons.

Resighting efforts and earlier detailed analyses of shag movements provided clear criteria for classifying surveyed sites into appropriate resident and migrant states (Acker, Burthe et al. 2021; Acker, Daunt et al. 2021; Ugland et al. 2024). Individuals staying resident on IoM are commonly observed to use day-roost sites along the Fife coastline (Figure S2) while returning to the IoM to roost for the night. Individuals observed on IoM and at Fife day-roost sites were therefore classified as resident (R). Individuals observed in Lothian are classified as migrant (M) as they do not return to IoM to roost and experience differing environmental conditions from residents. Similarly, all individuals observed at locations beyond IoM and the Fife sites are classified as migrant. To account for heterogeneity in detection between migrant locations with high- and low-intensity observations the migrant state was split into two sub-states: M1 (i.e., Lothian, Fraserburgh, Peterhead, Portknockie, and North Sutor) and M2 (all other locations) respectively (Figure S2). The definition of M1 and M2 was solely to account for differences in detection probability. Hence, there was no expectation for there to be any difference in movement or survival probabilities between these two sub-states. Consequently, key survival and movement parameters are jointly estimated across both migrant states (Figure S2; Supplementary Material S3). These states determine the classification for the encounter histories required to model movement and survival probabilities between consecutive winters.

Accordingly, we constructed encounter histories for all 9,358 colour-ringed individuals fledged in the 11 focal cohorts, each comprising a fledging occasion, four subsequent winter occasions, and a final ‘ever after’ occasion. This provided sufficient occasions to quantify sub-adult dynamics of partial migration, while avoiding too imprecise parameter estimation, as would occur through subsequent occasions following substantial cohort mortality. As locations during intervening summer months were not of direct interest for our current questions, summer resightings were not utilized. The numbers of shag chicks that were colour-ringed in each cohort, and hence appeared in each cohort-specific encounter history, are shown in Table S1.

**Table S1** Numbers of individual chicks colour-ringed in each cohort. Among-year variation in the numbers of ringed chicks reflects variation in the number of breeding pairs and breeding success, reflecting variable environmental conditions before and during the breeding season.

| **Cohort** | **Numbers of individual chicks ringed** |
| --- | --- |
| 2010 | 1101 |
| 2011 | 1125 |
| 2012 | 1038 |
| 2013 | 551 |
| 2014 | 755 |
| 2015 | 865 |
| 2016 | 802 |
| 2017 | 803 |
| 2018 | 711 |
| 2019 | 641 |
| 2020 | 966 |

During the sub-adult period numerous juveniles are not observed at any of the high-intensity sites in winter. As detection probability in the M2 state is expected to be very small, it effectively provides a ‘ghost-site’ for alive individuals that moved to unobserved locations during the sub-adult period. Further, the typically short distance natal dispersal (<4% of surviving individuals bred >50km from IoM, Barlow et al. 2013) coupled with high-intensity annual monitoring of individuals breeding on IoM and surrounding colonies means that any surviving individuals that temporarily emigrated from the observed system throughout the focal winters will likely be detected subsequently and hence appear as observed in the final ‘ever after’ occasion. Accordingly, estimated apparent survival probabilities will be close to the true survival probabilities. The ‘ever after’ occasion also acts as a ‘dummy’ occasion to ensure that all parameters up to occasion five can be fully estimated, as they would otherwise be non-identifiable in the final timestep of fully time-dependent MS-CMR models (Lebreton et al. 1992).

Each encounter history occasion comprises one datum for each individual. Multiple observations of the same individual within a single occasion were consequently collapsed into one. This was straightforward when all observations were in the same state. Across all cohorts and occasions there were 407 conflicts in 37,432 possible observation events where an individual was observed in more than one state within one occasion (1.1% of focal observations). These conflicts were mainly caused by individuals that were observed at or near IoM (i.e., as a resident) early within an occasion and then later observed at a migrant site (i.e., as a migrant) within the same occasion. Accordingly, for conflicts between R and either M state individuals were assigned to the corresponding M state to account for migrants that had either migrated late or returned early, and for conflicts between the two M states individuals were assigned to the M1 state. All resightings in the ‘ever after’ occasion were set to be resident regardless of location, effectively representing a global state. This simplifies the structure of the final ‘dummy’ occasion and associated parameters, while ensuring all parameters of interest are still identifiable. While MS-CMR models fundamentally assume instantaneous occasions (i.e., no mortality or movement within occasions), inferences are robust to violations when longer occasions such as ours are utilised, given approximately constant detection, movement, and survival within occasions (O’Brien, Robert, & Tiandry 2005).


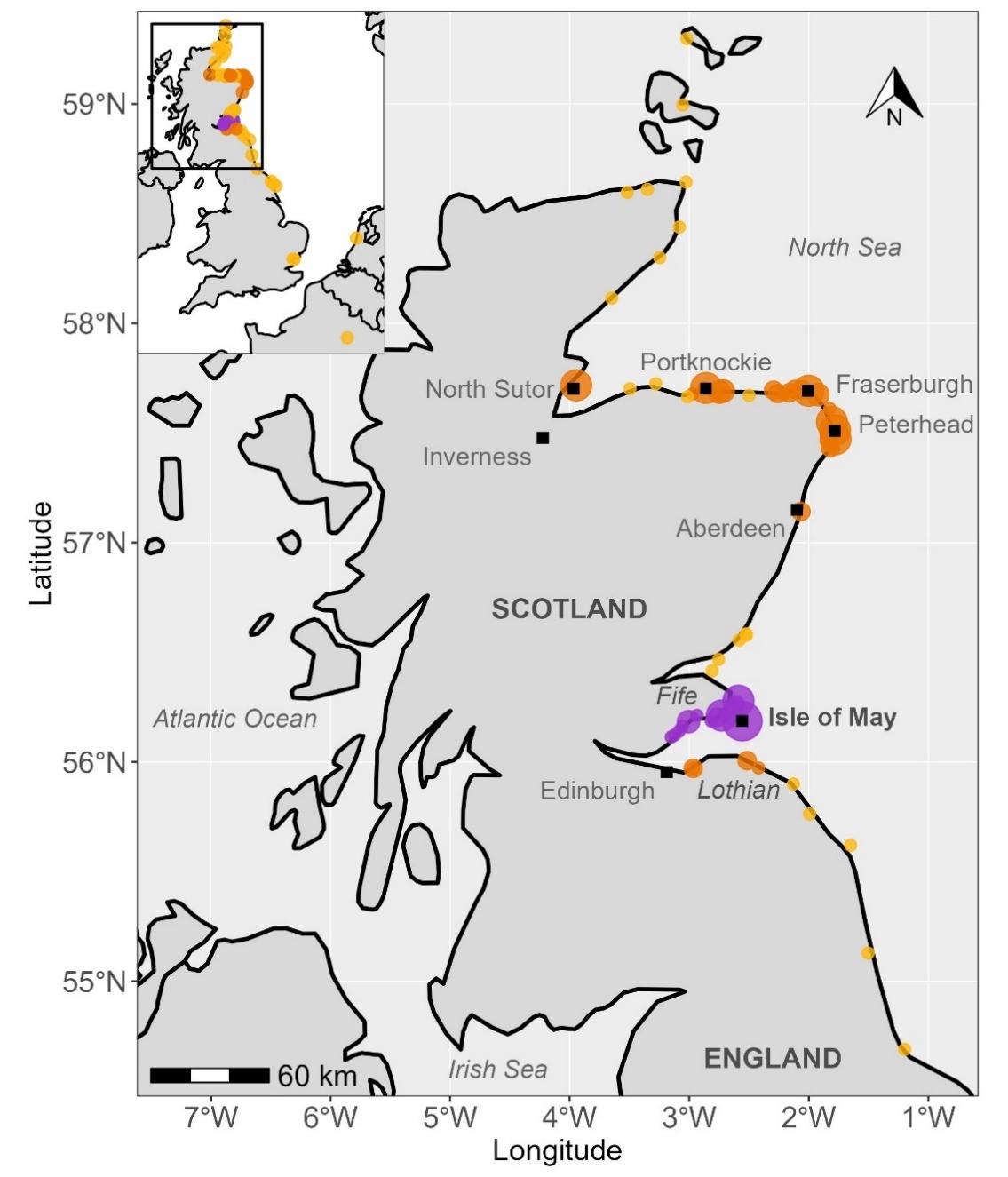


**Figure S2** Map of the study system resighting locations, with winter observations of individuals fledged on the Isle of May between 2010-2020 in the resident (R; purple points), intensively surveyed migrant (M1; orange points) and non-intensively surveyed migrant (M2; yellow points) states. Wider geographical resighting observations are shown on the top left, including observations from Southern England and continental Europe. The size of each point on the main map corresponds to the number of sightings at each location.

S3. Details of full model structure and parameters

Here we provide additional details of the full MS-CMR model structure including additional underlying movement and detection parameters that are structurally necessary and explanation of the M2 prior choice.

Our MS-CRM model structure estimates survival, movement, and detection probabilities involving four states: alive in the resident state (R), alive in migrant states 1 and 2 (M1 and M2 reflecting high and low surveying intensity respectively), and dead (D, Figure S3). Since we define two migrant states (M1 and M2) to handle spatial heterogeneity in detection probability (Figure S4, Supplementary Material S2) we require two additional movement parameters to model movement between the three alive states (Figure S3b; Ugland et al. 2024). The first parameter is the probability of transitioning to the M1 state conditional on departing from the resident state (*δ* conditional on *ε*). Accordingly, 1-*δ* is the probability of transitioning to the M2 state conditional on departing from the resident state. The second parameter is the probability that an individual in either migrant state switches (*γ*), or does not switch (1-*γ*), to the other migrant state. *γ* in either direction (i.e., M1 to M2 or M2 to M1) was assumed to be equal, as there was no expectation that individuals in one migrant state would be more likely to switch than the other, and *γ* is anyway estimated to be small.

Subjective prior choice for the M2 detection was based on known data structure (i.e., that detection must be low at sites that are not routinely surveyed). However, we note that the informativity of our dataset was such that this prior happened to have very little influence on the estimation of biological parameters of focal interest to our present study. Indeed, sensitivity analyses showed that posterior mean estimates of survival and movement probabilities were virtually identical when models were rerun with different priors on detection probability for the M2 migrant state (e.g., U[0,1], Beta[1,6] or Beta[0.025,6] compared with our chosen prior of Beta[0.5,6]). Nonetheless, our prior choice reduced uncertainty in estimates of M2 detection probability, which in turn slightly reduced uncertainty in estimates of migrant movement and survival probabilities (assumed equal across M1 and M2).

Overall, this model design handles all known major forms of structure in the data, including cohorts and occasions as well as differences in detection probabilities between the M1 and M2 sites and states. All key parameters of primary biological interest are explained in main Table 1. All nuisance parameter estimates are archived in the Dryad repository alongside key parameter estimates:

<http://datadryad.org/share/mjUGTHglCKSv5aWBHfdq7FgO5ALFQaMkEdLM3an6Odc>.


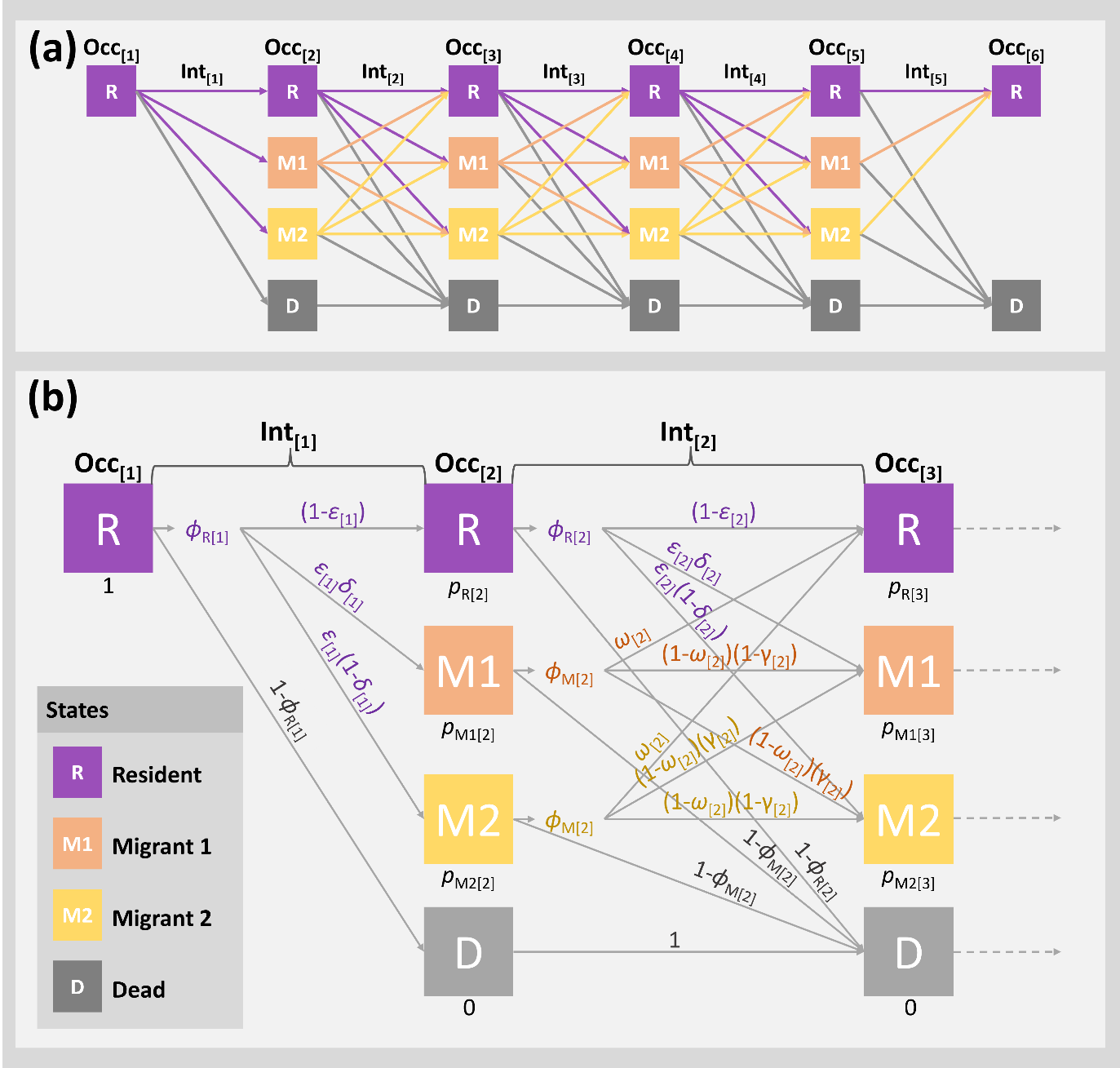


**Figure S3** Summary of (a) the full occasion (Occ), interval (Int), and transition (arrows) structure of the MS-CMR model and (b) details of the probabilities of transitioning between states (Resident: R, Migrant 1: M1, Migrant 2: M2, Dead: D, as defined in Supplementary Material S2) between the first to third occasions through the corresponding first and second intervals. The probabilities comprise survival probabilities (*ϕ*_R_, *ϕ*_M_), movement probabilities (*ε, ω, δ, γ*), and detection probabilities (*p*_R_*, p*_M1_*, p*_M2_), and are all indexed by the starting occasion. The additional migrant state (M2), required to account for known spatial heterogeneity in detection, generates the associated movement parameters *δ* and *γ*. Here, *δ* is the probability of transitioning to either the M1 (δ) or M2 (1- δ) state, conditional on having departed the resident state (*ε*), and *γ* is the probability of switching (γ) or not switching (1- γ) between the other migrant state, conditional on not having returned to the resident state (1-*ω*). Transitions between the dead state (D) are set to one, because it is not possible to be alive after being dead. The probabilities of being detected in the R, M1, and M2 states are *p*_R_*, p*_M1_*,* and *p*_M2_ respectively, and accordingly the probability of not being detected in each state is 1-*p*_R_*,* 1-*p*_M1_*,* and 1-*p*_M2_ respectively. The probability of being detected as resident in the first occasion is one when all individuals were ringed, while the probability of being detected in the D state is zero, as only live resightings were used. Analogous transitions occur until the fifth occasion as shown on panel (a), after which the structure is simplified for the sixth ‘ever after’ occasion where only survival is estimated. For further explanations of key parameters see Table 1.


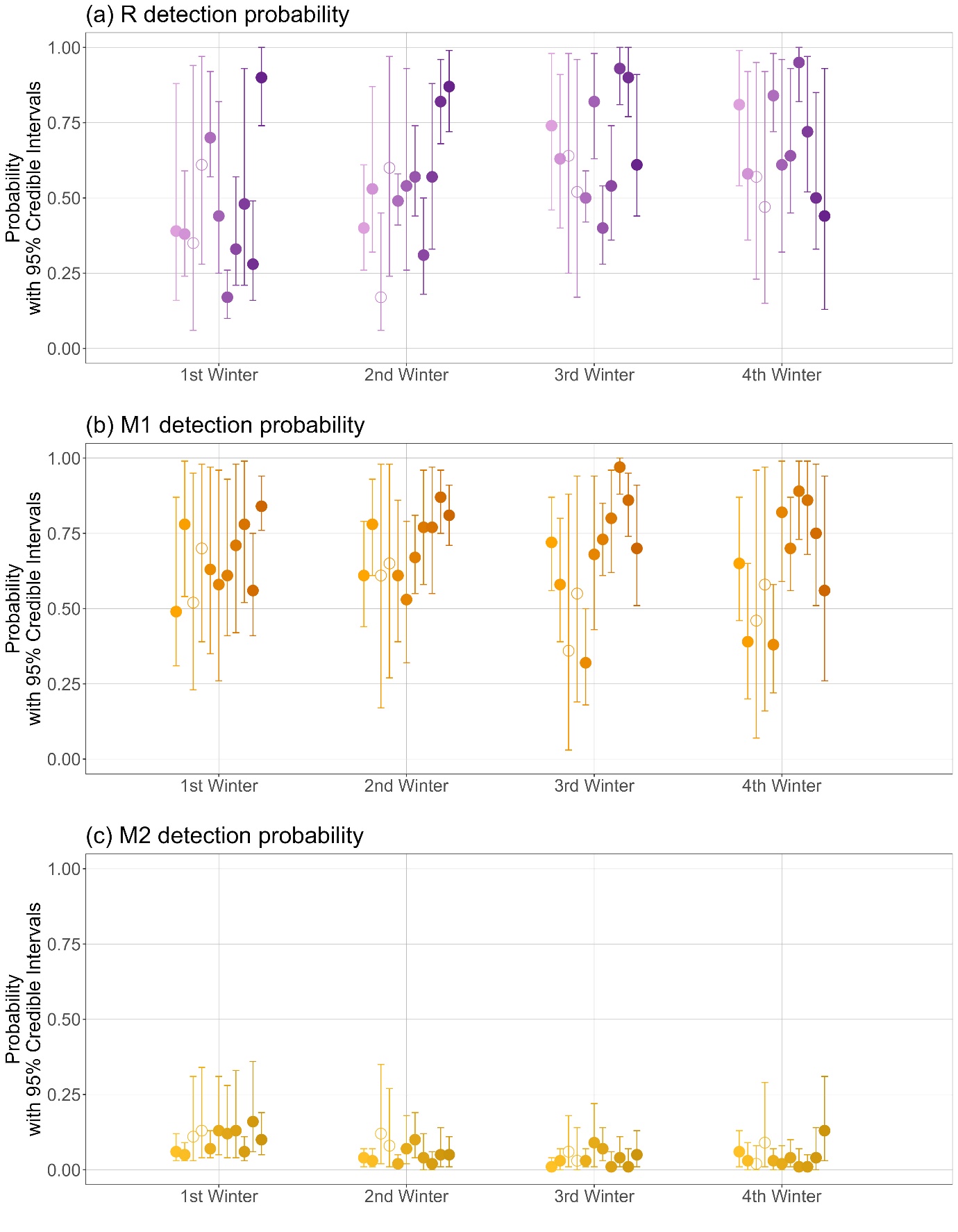


**Figure S4** Occasion specific detection probabilities for the (a) resident; R (b) intensively surveyed migrant; M1 and (c) non-intensively surveyed migrant; M2 states. Coloured points and lines show the posterior means for each cohort (2010-2020, light-dark) with 95% credible intervals (95%CI).

S4. Details of model checks

The fundamental validity of our MS-CMR model structure and resulting inferences has been previously demonstrated, by fitting the model to simulated datasets designed to be analogous to previous analyses of a different subset of the European shag dataset comprising multiple occasions through the first autumn and winter following fledging (Ugland et al. 2024). Simulated values were readily retrieved, with no evident bias. To further validate the model in the context of our current dataset and questions, we implemented posterior predictive checks (PPCs) to ensure that the observed encounter histories did not substantially differ from simulated encounter histories derived from our fitted models (Acker, Burthe et al. 2021; Ugland et al. 2024). These PPCs provide an overarching assessment of the degree to which the estimated model parameters can regenerate the observed data. Lack of congruence could indicate diverse underlying problems, including failure of the model to capture major forms of structure or heterogeneity in the data, or model coding errors.

Specifically, using the estimated model parameters for each cohort we simulated 16,000 independent samples to generate posterior distributions for encounter histories, assuming N released individuals, where N is the number of colour-ringed individuals in each focal cohort (Table S1). This generated posterior distributions for the frequencies (i.e., number) of individuals observed as resident, or as migrants in the intensively and non-intensively surveyed sites (M1 and M2 respectively), or unobserved in each occasion for each cohort. Accordingly, in the first occasion all N individuals are observed as resident. Subsequently, the estimated state-specific survival, movement, and detection probabilities are used to simulate numbers of individuals observed in each detection state, where the numbers of individuals in each occasion sum to N. We then checked whether the 95% credible intervals for the posterior distributions of the simulated frequencies encompassed the corresponding actual counts of observed residents, migrants, and unobserved individuals in the encounter history for each occasion and cohort. Note that, by construction, these PPCs concern the numbers of individuals that were actually and hypothetically observed in each state in each occasion. They do not directly concern the numbers of individuals that were actually present in each state in each occasion, which could be inferred from the model estimates but not directly assessed from the encounter history data (due to the detection failure).

These PPCs showed that the actual counts of observed individuals fall well within the 95%CIs of predicted counts for all cohorts and occasions (e.g., Figure S5, showing a sample of the 11 cohorts). This indicates good model fit, with no major bias in parameter estimates, or hence major underlying problems with unmodelled structure, heterogeneity, or misspecification.


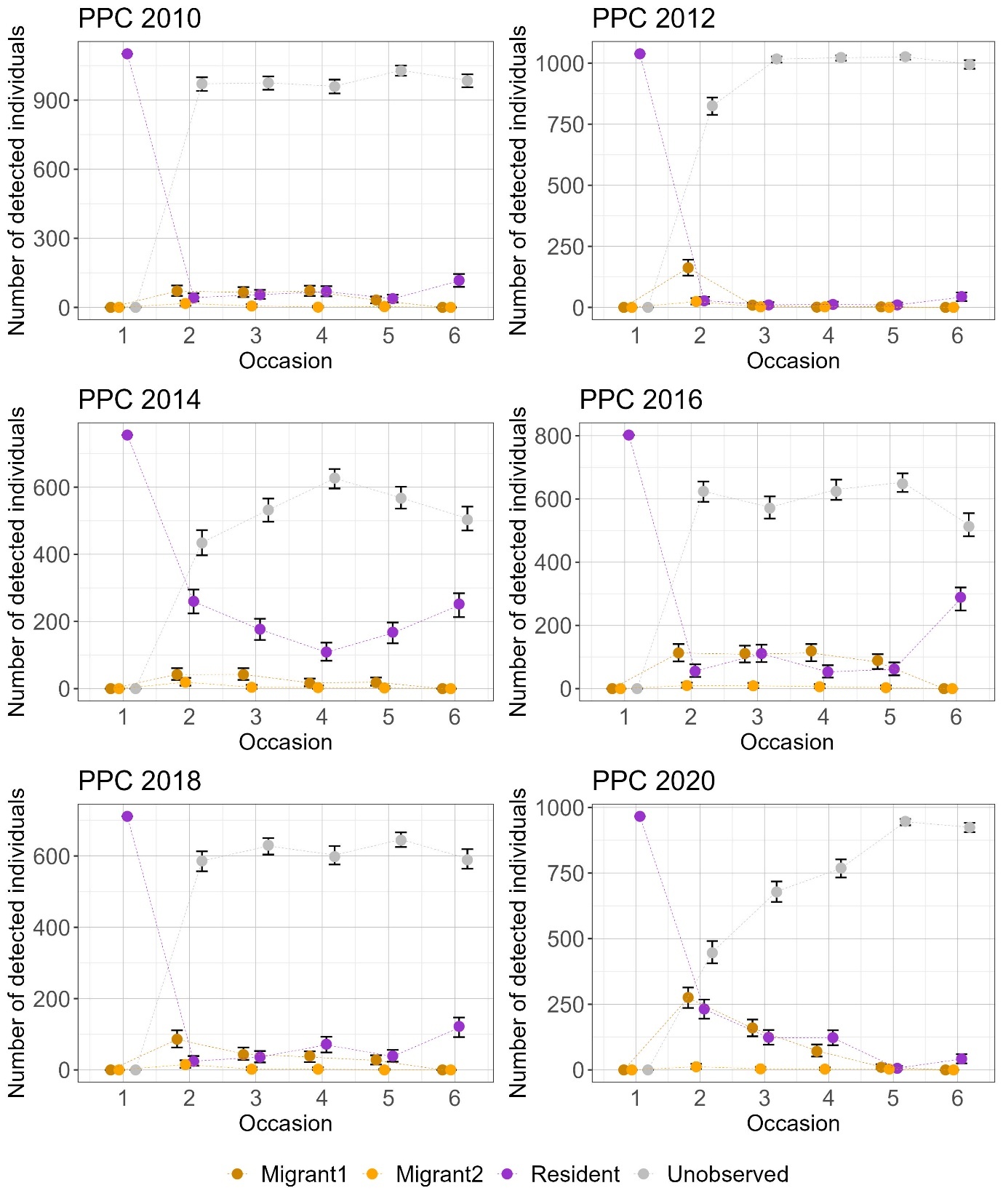
**Figure S5** Examples of posterior predictive checks (PPCs), showing numbers of observed (coloured points) and predicted (95% credible intervals and coloured lines) individuals per occasion for the 2010, 2012, 2014, 2016, 2018, and 2020 cohorts as visual examples. Coloured lines and points indicate the frequencies of observed and predicted individuals in each detection state (dark orange: migrant 1, light orange: migrant 2, purple: resident, and grey: unobserved).

S5. Non-consecutive winter repeatability

Our main analyses consider transitions, and hence phenotypic repeatability, between consecutive winters. Specifically, they estimate the expected proportions of individuals that remained in the same state between consecutive winters among all surviving individuals (*P*_S_). However, it is also of interest to quantify *P*_S_ between non-consecutive winters, representing repeatability across longer timeframes. For example, this approach could directly quantify the proportions of individuals that are in the same state in their 3^rd^ or 4^th^ winters as in their 1^st^ winter.

To achieve this, we refitted our MS-CMR model to three forms of reduced encounter history data, where we: i) dropped the 3^rd^ occasion (i.e., the 2^nd^ winter) and hence directly estimated transition probabilities between the 1^st^ and 3^rd^ winters; ii) dropped the 3^rd^ and 4^th^ occasions (i.e., the 2^nd^ and 3^rd^ winters) and hence directly estimated transition probabilities between the 1^st^ and 4^th^ winters; and iii) dropped the 4^th^ occasion (i.e., the 3^rd^ winter) and hence directly estimated transition probabilities between the 2^nd^ and 4^th^ winters. The expected proportions of individuals that stayed in the same state between non-consecutive winters were then calculated as for the main analyses of consecutive winters (since the reduced encounter histories mean that the focal non-consecutive winters are modelled consecutively).

These results show that P_S_ was almost as high between non-consecutive winters (with and without the 2012 and 2013 cohorts; Tables S2-3, Figure S6) as between consecutive winters (main Figure 3). Further, these results imply that most individuals typically already express their longer-term phenotype (migrant or resident) in their first winter.

These values are likely higher than would be expected if the estimated probabilities of resident-to-migrant and migrant-to-resident transitions between consecutive winters (Figure 2 in main text) were sequentially enacted randomly across individuals. Rather, they may imply some non-random individual plasticity, for example where individuals that switch phenotype between their first and second winters then switch back again by their third or fourth (and hence appear in the same phenotype across non-consecutive winters). Such individual effects are not explicitly quantified by our current analyses.

**Table S2** Cross-cohort posterior means of the (a) means and (b) variances with 95% credible interval (CI) limits for the proportions of surviving individuals that remained in the same state (P_S_) between non-consecutive winters for all cohorts, for each set of non-consecutive winters (1^st^-3^rd^ = [1_3], 1^st^-4^th^ = [1_4], 2^nd^-4^th^ = [2_4]).

|  | (a) Mean | | | (b) Variance | | |
| --- | --- | --- | --- | --- | --- | --- |
|  | Mean | 95% CI Limits | | Mean | 95% CI Limits | |
| Parameter |  | Lower | Upper |  | Lower | Upper |
| P_S[1_3]_ | 0.68 | 0.62 | 0.74 | 0.02 | 0.01 | 0.05 |
| P_S[1_4]_ | 0.65 | 0.58 | 0.72 | 0.03 | 0.01 | 0.05 |
| P_S[2_4]_ | 0.73 | 0.68 | 0.79 | 0.03 | 0.01 | 0.05 |

**Table S3** Cross-cohort posterior means of the (a) means and (b) variances with 95% credible interval (CI) limits for the proportions of surviving individuals that remained in the same state (P_S_) between non-consecutive winters for all cohorts excluding 2012 and 2013, for each set of non-consecutive winters (1^st^-3^rd^ = [1_3], 1^st^-4^th^ = [1_4], 2^nd^-4^th^ = [2_4]).

|  | (a) Mean | | | (b) Variance | | |
| --- | --- | --- | --- | --- | --- | --- |
|  | Mean | 95% CI Limits | | Mean | 95% CI Limits | |
| Parameter |  | Lower | Upper |  | Lower | Upper |
| P_S[1_3]_ | 0.71 | 0.66 | 0.75 | 0.01 | 0.00 | 0.02 |
| P_S[1_4]_ | 0.69 | 0.62 | 0.74 | 0.01 | 0.00 | 0.03 |
| P_S[2_4]_ | 0.77 | 0.72 | 0.81 | 0.01 | 0.00 | 0.02 |

**
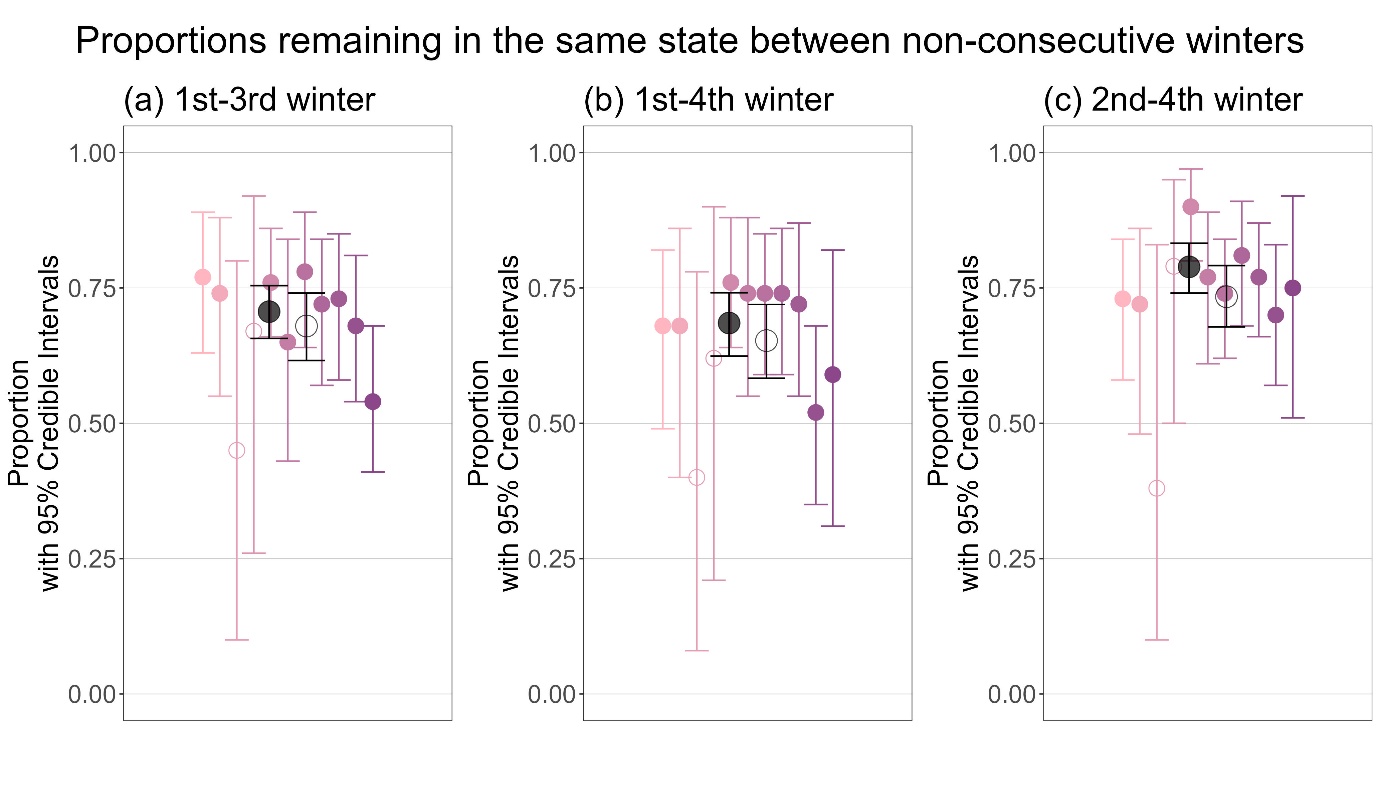
**

**Figure S6** Expected proportions of individuals that are in the same state between the (a) 1^st^- 3^rd^, (b) 1^st^-4^th^, and (c) 2^nd^-4^th^ winters, of all alive individuals. Coloured points and lines show the posterior mean for each cohort (2010-2020, light-dark) with 95% credible intervals (95%CI). Large black points and lines show the posterior means and 95%CIs across all cohorts combined (open points) and excluding 2012-2013 (filled points).

S6. Relationships between initial m and subsequent movement parameters

To quantify the degrees of reinforcing and compensatory movements following initial expression of migration versus residence we regressed and correlated the initial *m* (and hence initial *ε*, Supplementary Material S5) against all subsequent occasion-specific migratory fractions *m*, and interval-specific probabilities of departing from *ε* and returning to *ω* the resident state, across cohorts. Specifically, we computed the posterior distributions of the intercept and slope from simple linear regressions and Spearman’s rank correlations of each of these parameters against initial *m* (i.e., *m*_[2]_) across all cohorts to generate posterior mean regressions (Tables S4-5) and correlations (Tables S6-7) with 95%CIs. There was evidence for a positive relationship when excluding the 2012 and 2013 cohorts between initial *m* and 2^nd^-winter *m* (Figure S7a), showing that cohorts that are relatively migratory in the 1^st^ winter are also relatively migratory in the 2^nd^ winter, and vice versa. However, there was no evidence for a relationship between initial *m* and either the 3^rd^- or 4^th^-winter *m* (Tables S4-7; Figure S7), showing that cohorts that are relatively migratory in the 1^st^ winter are not necessarily so by the 3^rd^ winter or beyond. There was also no evidence for any strong relationship between initial *m* and either *ε* or *ω* for any interval, and hence no evidence for either reinforcing or compensatory movements associated with initial *m* (Tables S4-5; Figure S7).

**Table S4** Cross-cohort posterior means of the (a) slopes and (b) intercepts with 95% credible interval (CI) limits for the migratory fraction *m*, probabilities of departing from *ε* and returning to the resident state *ω* all cohorts, for each focal starting occasion [t].

|  | (a) Slope | | | (b) Intercept | | |
| --- | --- | --- | --- | --- | --- | --- |
|  | Mean | 95% CI Limits | | Mean | 95% CI Limits | |
| Parameter |  | Lower | Upper |  | Lower | Upper |
| *m*_[3]_ | 0.21 | -0.18 | 0.64 | 0.44 | 0.18 | 0.67 |
| *m*_[4]_ | 0.19 | -0.15 | 0.50 | 0.45 | 0.26 | 0.65 |
| *m*_[5]_ | 0.07 | -0.27 | 0.40 | 0.55 | 0.35 | 0.75 |
| *ε*_[2]_ | -0.01 | -0.49 | 0.56 | 0.26 | -0.07 | 0.56 |
| *ε*_[3]_ | 0.25 | -0.37 | 0.76 | 0.10 | -0.22 | 0.49 |
| *ε*_[4]_ | 0.02 | -0.44 | 0.49 | 0.24 | -0.04 | 0.53 |
| *ω*_[2]_ | 0.26 | -0.28 | 0.67 | 0.09 | -0.16 | 0.43 |
| *ω*_[3]_ | 0.27 | -0.18 | 0.72 | 0.09 | -0.18 | 0.37 |
| *ω*_[4]_ | 0.15 | -0.15 | 0.52 | 0.10 | -0.11 | 0.29 |

**Table S5** Cross-cohort posterior means of the (a) slopes and (b) intercepts with 95% credible interval (CI) limits for the migratory fraction *m*, probabilities of departing from *ε* and returning to the resident state *ω* all cohorts excluding 2012 and 2013, for each focal starting occasion [t].

|  | (a) Slope | | | (b) Intercept | | |
| --- | --- | --- | --- | --- | --- | --- |
|  | Mean | 95% CI Limits | | Mean | 95% CI Limits | |
| Parameter |  | Lower | Upper |  | Lower | Upper |
| *m*_[3]_ | 0.45 | 0.15 | 0.74 | 0.32 | 0.13 | 0.51 |
| *m*_[4]_ | 0.16 | -0.12 | 0.46 | 0.46 | 0.28 | 0.64 |
| *m*_[5]_ | 0.07 | -0.26 | 0.39 | 0.55 | 0.35 | 0.74 |
| *ε*_[2]_ | -0.16 | -0.57 | 0.27 | 0.32 | 0.07 | 0.58 |
| *ε*_[3]_ | -0.10 | -0.46 | 0.27 | 0.25 | 0.03 | 0.47 |
| *ε*_[4]_ | 0.02 | -0.46 | 0.50 | 0.23 | -0.05 | 0.53 |
| *ω*_[2]_ | -0.05 | -0.39 | 0.24 | 0.22 | 0.04 | 0.45 |
| *ω*_[3]_ | 0.10 | -0.25 | 0.45 | 0.16 | -0.05 | 0.37 |
| *ω*_[4]_ | 0.18 | -0.11 | 0.50 | 0.07 | -0.11 | 0.25 |

**Table S6** Cross-cohort posterior means of the Spearman’s rank correlations with 95% credible interval (CI) limits for the migratory fraction *m*, probabilities of departing from *ε* and returning to the resident state *ω* all cohorts, for each focal starting occasion [t].

|  | Mean | 95% CI Limits | |
| --- | --- | --- | --- |
| Parameter |  | Lower | Upper |
| *m*_[3]_ | 0.36 | -0.06 | 0.80 |
| *m*_[4]_ | 0.28 | -0.18 | 0.64 |
| *m*_[5]_ | 0.10 | -0.35 | 0.55 |
| *ε*_[2]_ | -0.10 | -0.65 | 0.47 |
| *ε*_[3]_ | 0.11 | -0.36 | 0.54 |
| *ε*_[4]_ | 0.02 | -0.45 | 0.51 |
| *ω*_[2]_ | 0.14 | -0.38 | 0.59 |
| *ω*_[3]_ | 0.27 | -0.16 | 0.65 |
| *ω*_[4]_ | 0.27 | -0.23 | 0.69 |

**Table S7** Cross-cohort posterior means of the Spearman’s rank correlations with 95% credible interval (CI) limits for the migratory fraction *m*, probabilities of departing from *ε* and returning to the resident state *ω* all cohorts excluding 2012 and 2013, for each focal starting occasion [t].

|  | Mean | 95% CI Limits | |
| --- | --- | --- | --- |
| Parameter |  | Lower | Upper |
| *m*_[3]_ | 0.68 | 0.25 | 0.93 |
| *m*_[4]_ | 0.23 | -0.20 | 0.62 |
| *m*_[5]_ | 0.09 | -0.40 | 0.58 |
| *ε*_[2]_ | -0.22 | -0.75 | 0.43 |
| *ε*_[3]_ | -0.13 | -0.63 | 0.42 |
| *ε*_[4]_ | 0.04 | -0.50 | 0.57 |
| *ω*_[2]_ | -0.09 | -0.63 | 0.48 |
| *ω*_[3]_ | 0.18 | -0.30 | 0.63 |
| *ω*_[4]_ | 0.36 | -0.18 | 0.77 |


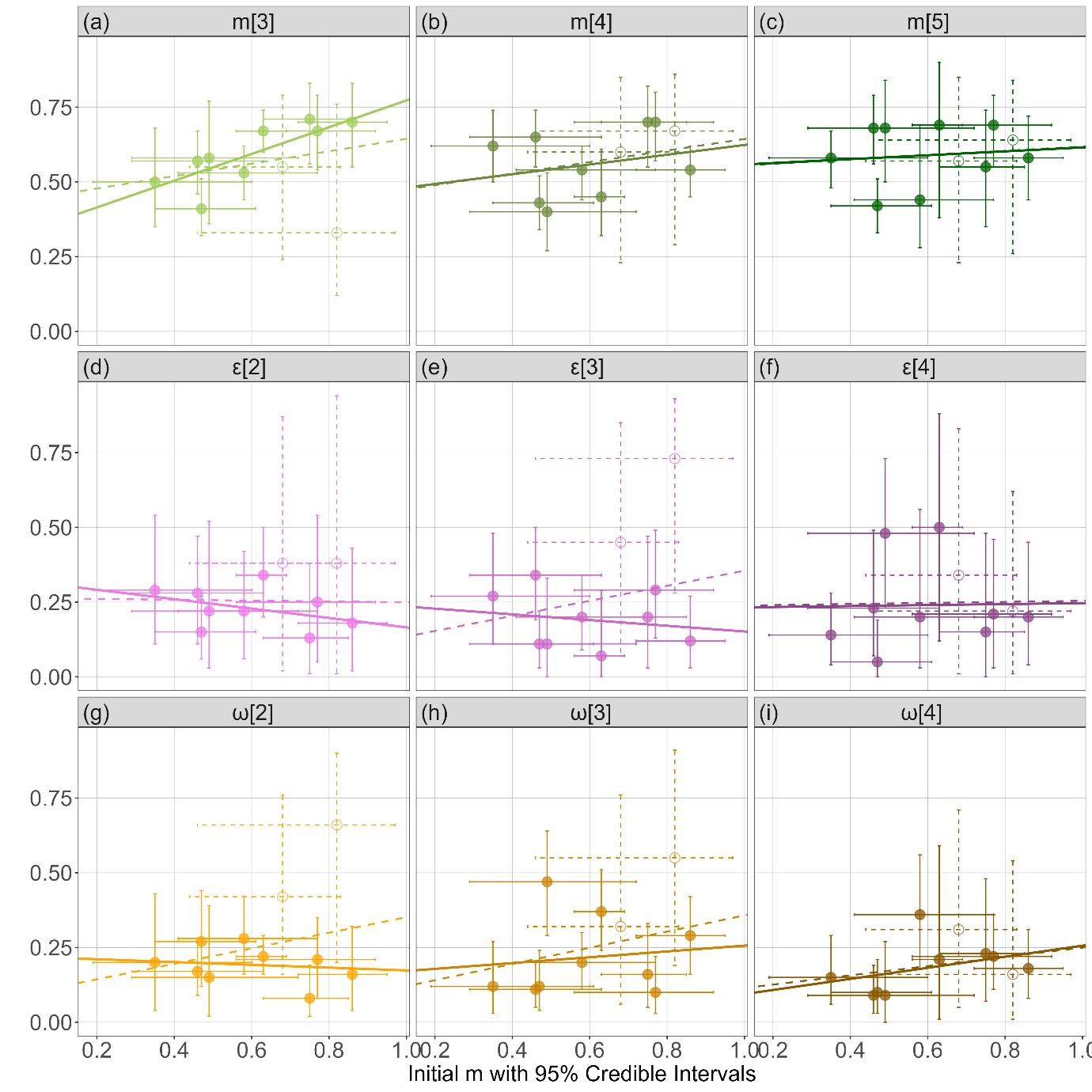


**Figure S7** Linear regressions between initial departure (i.e., initial migratory fraction, *m*_[2]_) following fledging and all subsequent migratory fractions (*m*; a-c), departure (*ε*; d-f), and return (*ω*; g-i) probabilities. Coloured points indicate the posterior mean estimates for each cohort with 95% credible intervals (95%CI). Solid lines show posterior mean regressions for each pair of movement parameters for all cohorts excluding 2012-2013, while dashed lines show posterior mean regressions for each pair of movement parameters for all cohorts including 2012-2013. 95% CIs for all regression slopes overlap 0 aside from the relationship between *m*_[2]_ and *m*_[3]_ when the 2012 and 2013 cohorts were excluded.

S7. Joint effects of plasticity and selection on partial migration

To test for systematic associations between the directions of selection and plasticity we quantified the degree to which change in the migratory fraction *m* without plasticity (i.e., due only to selection, *Δ_m.sel_*) predicted the magnitude and direction of change in the migratory fraction *m* without selection (i.e., due only to plasticity, *Δ_m.pl_*). This was achieved by calculating the full posterior distributions of the slopes and intercepts of the regressions between *Δ_m.sel_* and *Δ_m.pl_* with 95%CIs across cohorts and ages. Here a positive relationship would indicate that selection for or against migration was associated with increased or decreased migratory plasticity respectively, generating reinforcing effects. Conversely, a negative relationship would indicate that increased migration due to selection would be associated with decreases in migratory plasticity, and vice versa, diminishing net change in the degree of partial migration. We found no evidence for relationships between *Δ_m.sel_* and *Δ_m.pl_* across cohorts and ages as all 95%CIs overlapped zero (slopes with 2012 and 2013: -0.22[-1.54,1.09], -1.65[-3.91,0.50], 0.12[-0.99,1.17] and without 2012 and 2013: 0.22[-0.60,1.10], -1.00[-2.91,0.54], 0.19[-0.92,1.25] for the 1^st^-2^nd^, 2^nd^-3^rd^, and 3^rd^-4^th^ winters respectively; Figure S8a), indicating the form and magnitude of selection does not predict subsequent plasticity in either direction.

Further, we quantified the absolute difference between *Δ_m.pl_* and *Δ_m.sel_* for each cohort between consecutive winters, and hence ages, to determine whether plasticity had larger effects on overall change in *m* than selection, or vice versa. Here, values above or below zero indicate that the effects of plasticity or selection are larger respectively. There was no consistent evidence of either *Δ_m.sel_* or *Δ_m.pl_* exceeding the other, and accordingly no evidence for consistent effects of plasticity or selection contributing more to overall change in *m* (95% credible intervals for all cohorts across all ages overlapping zero apart from 2018 (0.15[0.01,0.29]) and 2020 (0.21[0.06,0.33]) in the 2^nd^-3^rd^ winter; Figure S8b; Table S8).

**Table S8** Cohort and interval [t] specific estimates of the posterior mean with 95% credible interval (CI) limits for the difference between *Δ_m.sel_* and *Δ_m.pl_* as presented on Figure S8b.

| Cohort interval | Mean | 95% CI limits | |
| --- | --- | --- | --- |
|  |  | Lower | Upper |
| 2010_[2]_ | 0.09 | -0.03 | 0.27 |
| 2010_[3]_ | 0.02 | -0.06 | 0.12 |
| 2010_[4]_ | 0.01 | -0.14 | 0.18 |
| 2011_[2]_ | 0.01 | -0.06 | 0.10 |
| 2011_[3]_ | 0.01 | -0.12 | 0.19 |
| 2011_[4]_ | 0.07 | -0.07 | 0.27 |
| 2012_[2]_ | 0.44 | -0.06 | 0.78 |
| 2012_[3]_ | 0.34 | -0.05 | 0.66 |
| 2012_[4]_ | 0.06 | -0.07 | 0.32 |
| 2013_[2]_ | 0.13 | -0.17 | 0.48 |
| 2013_[3]_ | 0.11 | -0.09 | 0.45 |
| 2013_[4]_ | 0.12 | -0.08 | 0.48 |
| 2014_[2]_ | 0.03 | -0.06 | 0.16 |
| 2014_[3]_ | 0.00 | -0.06 | 0.08 |
| 2014_[4]_ | 0.01 | -0.03 | 0.08 |
| 2015_[2]_ | -0.01 | -0.17 | 0.19 |
| 2015_[3]_ | 0.18 | -0.05 | 0.40 |
| 2015_[4]_ | 0.12 | -0.07 | 0.34 |
| 2016_[2]_ | 0.02 | -0.09 | 0.19 |
| 2016_[3]_ | 0.07 | -0.01 | 0.18 |
| 2016_[4]_ | 0.03 | -0.03 | 0.13 |
| 2017_[2]_ | 0.04 | -0.13 | 0.25 |
| 2017_[3]_ | 0.00 | -0.13 | 0.16 |
| 2017_[4]_ | 0.03 | -0.04 | 0.14 |
| 2018_[2]_ | 0.03 | -0.11 | 0.21 |
| 2018_[3]_ | 0.15 | 0.01 | 0.29 |
| 2018_[4]_ | -0.01 | -0.09 | 0.10 |
| 2019_[2]_ | 0.06 | -0.07 | 0.24 |
| 2019_[3]_ | 0.02 | -0.04 | 0.10 |
| 2019_[4]_ | 0.11 | -0.01 | 0.26 |
| 2020_[2]_ | -0.04 | -0.11 | 0.05 |
| 2020_[3]_ | 0.21 | 0.06 | 0.33 |
| 2020_[4]_ | -0.09 | -0.31 | 0.19 |


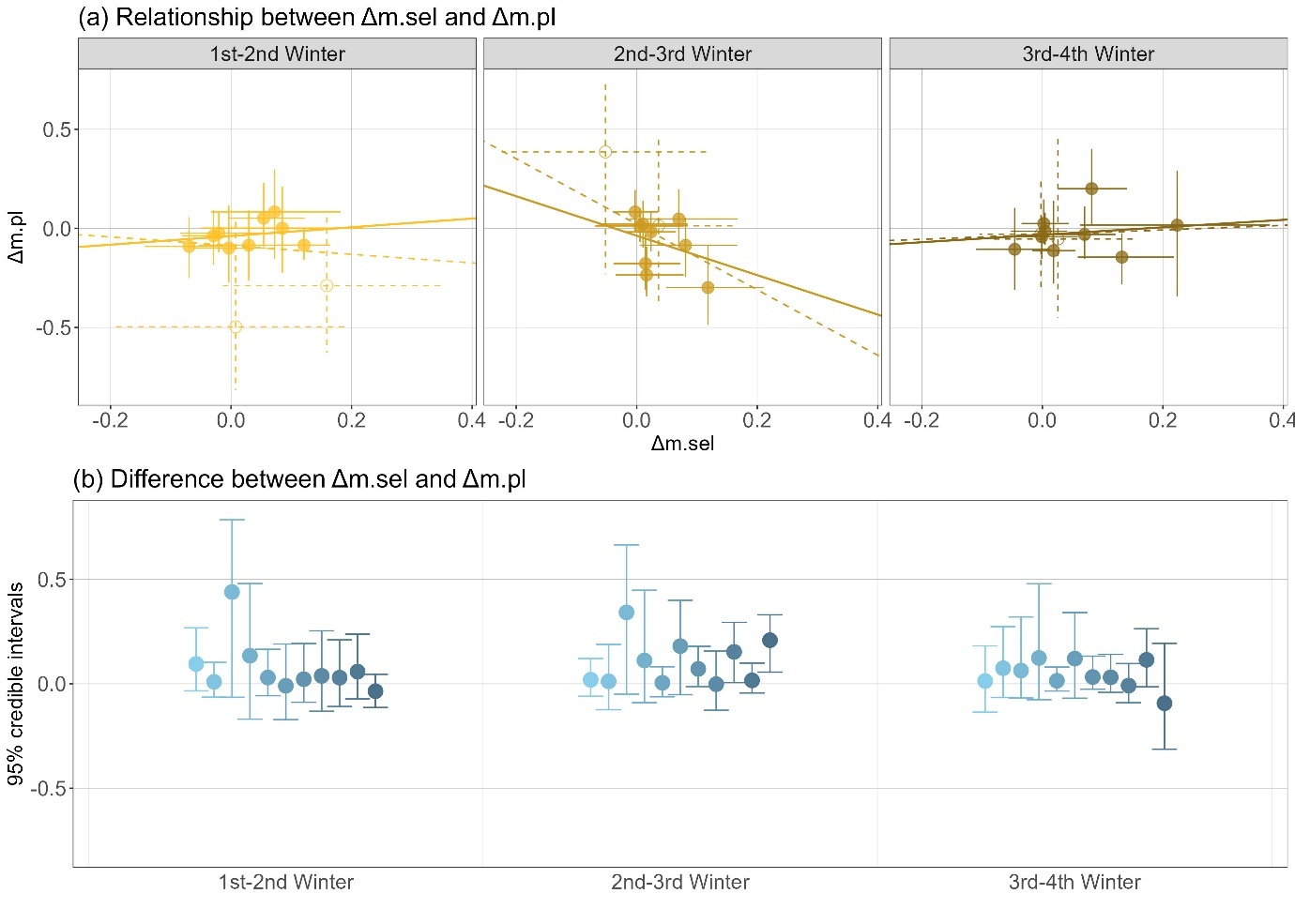


**Figure S8** (a) Linear regressions between the change in the migratory fraction *m* without plasticity (*Δ_m.sel_*) and the change in without selection (*Δ_m.pl_*) for each interval between consecutive winters. Solid lines show regressions for each pair of movement parameters for all cohorts excluding 2012-2013, while dashed lines show regressions for each pair of movement parameters for all cohorts including 2012-2013. 95% credible intervals (CI) for all regression slopes overlap 0. (b) Difference between (*Δ_m.pl_*) and (*Δ_m.sel_*) for each cohort and ages, where points and 95%CIs above and below zero indicate stronger effects of plasticity and selection respectively. Coloured point on both panels indicates the posterior mean estimates for each cohort with 95%CI.

S8. Cross-cohort movement results

Here we present the cross-cohort means and variances for key movement parameters both with and without the 2012 and 2013 cohorts. Tables S9 and S10 present the migratory fraction *m*_[_*_t_*_]_, and the probabilities of departing from *ε*_[_*_t_*_]_ and returning to the resident state *ω*_[_*_t_*_]_, the proportions of surviving individuals that remained in the same state P_S[_*_t_*_]_, moved from resident to migrant P_RM[_*_t_*_]_, and moved from migrant to resident P_MR[_*_t_*_]_ for the focal time steps. The cross-cohort means and variances are presented with posterior mean estimates and 95% credible intervals (CI).

**Table S9** Cross-cohort posterior means of the (a) means and (b) variances with 95% credible interval (CI) limits for the migratory fraction *m*, probabilities of departing from *ε* and returning to the resident state *ω*, the proportions of surviving individuals that: remained in the same state P_S_, moved from resident to migrant P_RM_, and moved from migrant to resident P_MR_ for all cohorts, for each focal starting occasion [t].

|  | (a) Mean | | | (b) Variance | | |
| --- | --- | --- | --- | --- | --- | --- |
|  | Mean | 95% CI Limits | | Mean | 95% CI Limits | |
| Parameter |  | Lower | Upper |  | Lower | Upper |
| *m*_[2]_ | 0.63 | 0.57 | 0.68 | 0.04 | 0.02 | 0.05 |
| *m*_[3]_ | 0.57 | 0.51 | 0.62 | 0.02 | 0.01 | 0.04 |
| *m*_[4]_ | 0.57 | 0.52 | 0.62 | 0.02 | 0.01 | 0.03 |
| *m*_[5]_ | 0.59 | 0.53 | 0.65 | 0.02 | 0.01 | 0.04 |
| *ε*_[2]_ | 0.26 | 0.18 | 0.34 | 0.03 | 0.01 | 0.07 |
| *ε*_[3]_ | 0.26 | 0.20 | 0.33 | 0.05 | 0.02 | 0.08 |
| *ε*_[4]_ | 0.25 | 0.17 | 0.34 | 0.04 | 0.01 | 0.08 |
| *ω*_[2]_ | 0.26 | 0.20 | 0.31 | 0.04 | 0.01 | 0.06 |
| *ω*_[3]_ | 0.26 | 0.20 | 0.32 | 0.03 | 0.01 | 0.07 |
| *ω*_[4]_ | 0.19 | 0.14 | 0.26 | 0.02 | 0.01 | 0.04 |
| P_S[2]_ | 0.74 | 0.69 | 0.79 | 0.03 | 0.01 | 0.05 |
| P_S[3]_ | 0.73 | 0.68 | 0.77 | 0.03 | 0.01 | 0.05 |
| P_S[4]_ | 0.78 | 0.73 | 0.83 | 0.01 | 0.00 | 0.03 |
| P_RM[2]_ | 0.08 | 0.06 | 0.12 | 0.00 | 0.00 | 0.01 |
| P_RM[3]_ | 0.13 | 0.08 | 0.17 | 0.03 | 0.00 | 0.05 |
| P_RM[4]_ | 0.09 | 0.06 | 0.14 | 0.01 | 0.00 | 0.03 |
| P_MR[2]_ | 0.18 | 0.13 | 0.23 | 0.03 | 0.01 | 0.06 |
| P_MR[3]_ | 0.15 | 0.11 | 0.19 | 0.01 | 0.00 | 0.03 |
| P_MR[4]_ | 0.12 | 0.08 | 0.17 | 0.01 | 0.00 | 0.02 |

**Table S10** Cross-cohort (a) means and (b) variances for the migratory fraction *m*, probabilities of departing from *ε* and returning to the resident state *ω*, the proportions of surviving individuals that: remained in the same state P_S_, moved from resident to migrant P_RM_, and moved from migrant to resident P_MR_ for all cohorts excluding 2012 and 2013, for each focal starting occasion [t].

|  | (a) Mean | | | (b) Variance | | |
| --- | --- | --- | --- | --- | --- | --- |
|  | Mean | 95% CI Limits | | Mean | 95% CI Limits | |
| Parameter |  | Lower | Upper |  | Lower | Upper |
| *m*_[2]_ | 0.60 | 0.54 | 0.65 | 0.04 | 0.02 | 0.06 |
| *m*_[3]_ | 0.59 | 0.55 | 0.64 | 0.02 | 0.01 | 0.03 |
| *m*_[4]_ | 0.56 | 0.52 | 0.60 | 0.02 | 0.01 | 0.03 |
| *m*_[5]_ | 0.59 | 0.53 | 0.64 | 0.02 | 0.01 | 0.03 |
| *ε*_[2]_ | 0.23 | 0.17 | 0.30 | 0.02 | 0.00 | 0.03 |
| *ε*_[3]_ | 0.19 | 0.14 | 0.25 | 0.02 | 0.01 | 0.03 |
| *ε*_[4]_ | 0.24 | 0.16 | 0.33 | 0.04 | 0.01 | 0.08 |
| *ω*_[2]_ | 0.19 | 0.15 | 0.24 | 0.01 | 0.00 | 0.02 |
| *ω*_[3]_ | 0.22 | 0.18 | 0.26 | 0.02 | 0.01 | 0.04 |
| *ω*_[4]_ | 0.18 | 0.13 | 0.24 | 0.02 | 0.00 | 0.04 |
| P_S[2]_ | 0.79 | 0.75 | 0.83 | 0.01 | 0.00 | 0.01 |
| P_S[3]_ | 0.78 | 0.75 | 0.82 | 0.01 | 0.00 | 0.02 |
| P_S[4]_ | 0.79 | 0.75 | 0.83 | 0.01 | 0.00 | 0.02 |
| P_RM[2]_ | 0.09 | 0.06 | 0.12 | 0.00 | 0.00 | 0.01 |
| P_RM[3]_ | 0.07 | 0.05 | 0.10 | 0.00 | 0.00 | 0.01 |
| P_RM[4]_ | 0.09 | 0.06 | 0.13 | 0.01 | 0.00 | 0.02 |
| P_MR[2]_ | 0.12 | 0.09 | 0.16 | 0.00 | 0.00 | 0.01 |
| P_MR[3]_ | 0.14 | 0.11 | 0.18 | 0.01 | 0.00 | 0.02 |
| P_MR[4]_ | 0.11 | 0.08 | 0.16 | 0.01 | 0.00 | 0.02 |

S9. Cross-cohort survival results

Here we present cohort-specific survival estimates (Figure S9) and the cross-cohort means and variances for key survival estimates both with and without the 2012 and 2013 cohorts. Tables S11 and S12 present the resident *ϕ*_R[_*_t_*_]_ and migrant *ϕ*_M[_*_t_*_]_ survival probabilities and the survival difference between migrants and residents *Δ_ϕ_*_[_*_t_*_]_ for the focal interval. The cross-cohort means and variances are presented with posterior mean estimates and 95% credible intervals (CI).

Extreme winter storms in 2012, 2013, and 2023 led to very high first-winter mortality (<0.2) for the 2012 and 2013 cohorts (Ugland et al. 2024), and third-winter mortality for the 2020 cohort. The 2012 storm also likely led to lower resident survival for the 2011 and 2010 cohorts in their second and third winters respectively. Such extreme climatic events occur periodically in the focal shag population (Acker, Daunt et al. 2021; Ugland et al. 2024).

**Table S11** Cross-cohort posterior means of the (a) means and (b) variances with 95% credible interval (CI) limits for the resident *ϕ*_R_ and migrant *ϕ*_M_ survival probabilities and the survival difference between migrants and residents *Δ_ϕ_* for all cohorts, for each focal interval [t].

|  | (a) Mean | | | (b) Variance | | |
| --- | --- | --- | --- | --- | --- | --- |
|  | Mean | 95% CI Limits | | Mean | 95% CI Limits | |
| Parameter |  | Lower | Upper |  | Lower | Upper |
| *ϕ*_R[1]_ | 0.74 | 0.69 | 0.78 | 0.02 | 0.01 | 0.03 |
| *ϕ*_R[2]_ | 0.49 | 0.45 | 0.54 | 0.10 | 0.07 | 0.13 |
| *ϕ*_R[3]_ | 0.67 | 0.61 | 0.73 | 0.03 | 0.02 | 0.05 |
| *ϕ*_R[4]_ | 0.68 | 0.63 | 0.72 | 0.07 | 0.06 | 0.09 |
| *ϕ*_M[2]_ | 0.52 | 0.49 | 0.55 | 0.07 | 0.06 | 0.09 |
| *ϕ*_M[3]_ | 0.76 | 0.71 | 0.81 | 0.03 | 0.02 | 0.06 |
| *ϕ*_M[4]_ | 0.76 | 0.71 | 0.81 | 0.05 | 0.03 | 0.06 |
| *Δ_ϕ_*_[2]_ | 0.03 | -0.03 | 0.08 | 0.04 | 0.02 | 0.06 |
| *Δ_ϕ_*_[3]_ | 0.09 | 0.01 | 0.16 | 0.04 | 0.01 | 0.07 |
| *Δ_ϕ_*_[4]_ | 0.09 | 0.02 | 0.15 | 0.03 | 0.01 | 0.06 |

**Table S12** Cross-cohort posterior means of the (a) means and (b) variances with 95% credible interval (CI) limits for the resident *ϕ*_R_ and migrant *ϕ*_M_ survival probabilities and the survival difference between migrants and residents *Δ_ϕ_* for all cohorts excluding 2012 and 2013, for each focal interval [t].

|  | (a) Mean | | | (b) Variance | | |
| --- | --- | --- | --- | --- | --- | --- |
|  | Mean | 95% CI Limits | | Mean | 95% CI Limits | |
| Parameter |  | Lower | Upper |  | Lower | Upper |
| *ϕ_R_*_[1]_ | 0.72 | 0.68 | 0.76 | 0.02 | 0.01 | 0.03 |
| *ϕ*_R[2]_ | 0.58 | 0.53 | 0.63 | 0.07 | 0.05 | 0.10 |
| *ϕ*_R[3]_ | 0.67 | 0.61 | 0.72 | 0.03 | 0.02 | 0.05 |
| *ϕ*_R[4]_ | 0.65 | 0.61 | 0.69 | 0.08 | 0.07 | 0.10 |
| *ϕ*_M[2]_ | 0.61 | 0.57 | 0.65 | 0.04 | 0.03 | 0.06 |
| *ϕ*_M[3]_ | 0.78 | 0.73 | 0.82 | 0.03 | 0.01 | 0.04 |
| *ϕ*_M[4]_ | 0.75 | 0.71 | 0.78 | 0.06 | 0.04 | 0.07 |
| *Δ_ϕ_*_[2]_ | 0.02 | -0.04 | 0.09 | 0.04 | 0.02 | 0.07 |
| *Δ_ϕ_*_[3]_ | 0.11 | 0.05 | 0.18 | 0.03 | 0.01 | 0.05 |
| *Δ_ϕ_*_[4]_ | 0.10 | 0.04 | 0.15 | 0.03 | 0.01 | 0.05 |


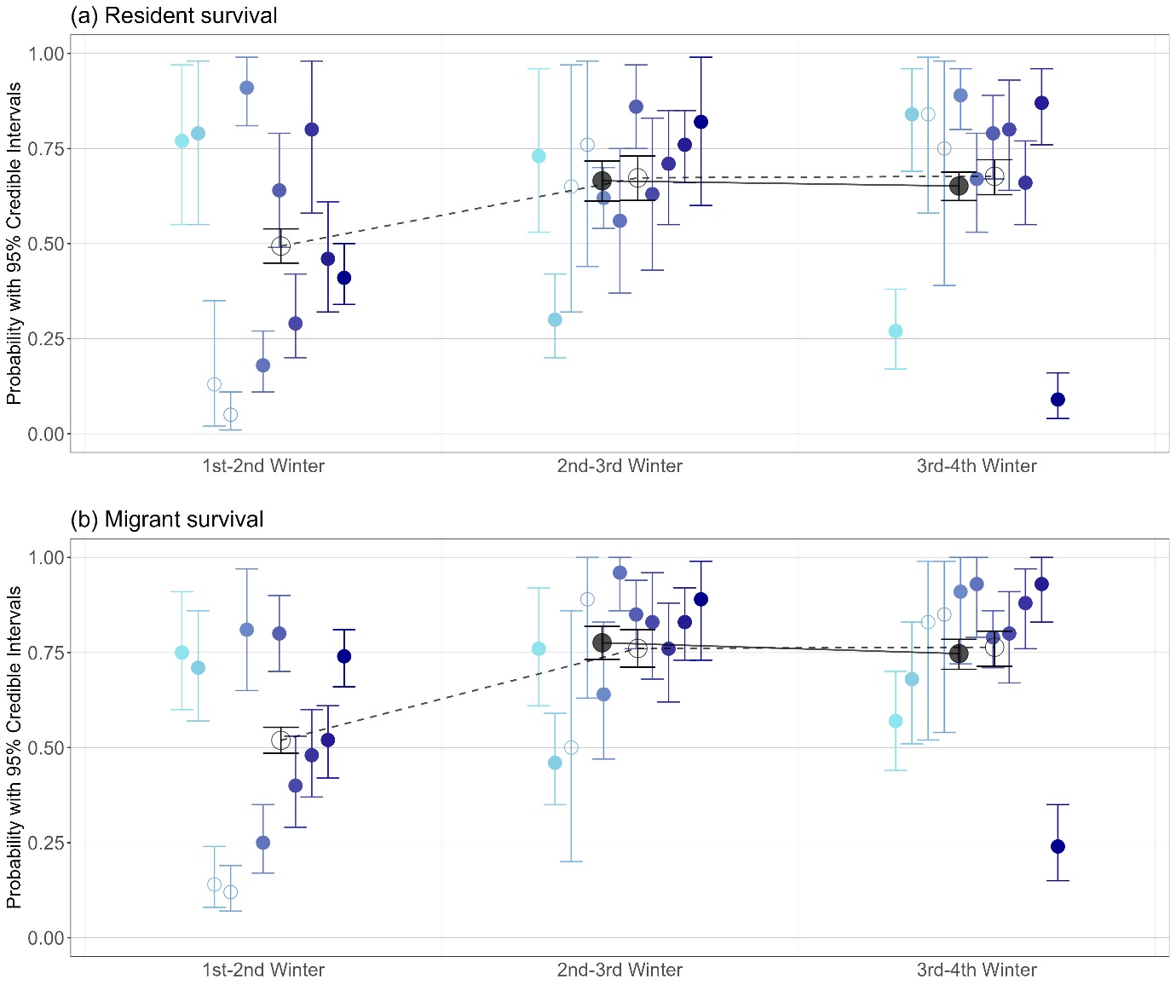


**Figure S9** Interval specific probabilities of (a) resident and (b) migrant survival. Coloured points and lines show the posterior means for each cohort (2010-2020, light-dark) with 95% credible intervals (95%CI). Large black points and lines show the posterior means and 95%CIs across all cohorts combined (open points) and excluding 2012-2013 (filled points).

References

Acker, P., Burthe, S.J., Newell, M.A., Grist, H., Gunn, C., Harris, M.P., Payo-Payo, A., Swann, R.L., Wanless, S., Daunt, F., Reid, J.M. (2021) Episodes of opposing survival and reproductive selection cause strong fluctuating selection on seasonal migration versus residence. *Proc. R. Soc. B* **288**(1951), 20210404. <https://doi.org/10.1098/RSPB.2021.0404>

Acker, P., Daunt, F., Wanless, S., Burthe, S.J., Newell, M.A., Harris, M.P., Grist, H., Sturgeon, J., Swann, R.L., Gunn, C., Payo-Payo, A., Reid, J.M. (2021) Strong survival selection on seasonal migration versus residence induced by extreme climatic events. *J. Anim. Ecol.* **90**(4), 796–808. <https://doi.org/10.1111/1365-2656.13410>

Aebischer, N.J., Potts, G.R., Coulson, J.C. (1995) Site and mate fidelity of Shags Phalacrocorax aristotelis at two British colonies. *Ibis*. **137**(1), 19–28. <https://doi.org/10.1111/j.1474-919X.1995.tb03215.x>

Barlow, E.J., Daunt, F., Wanless, S., Reid, J.M. (2013) Estimating dispersal distributions at multiple scales: Within-colony and among-colony dispersal rates, distances and directions in European Shags Phalacrocorax aristotelis. *Ibis* **155**(4), 762–778. <https://doi.org/10.1111/ibi.12060>

Grist, H., Daunt, F., Wanless, S., Nelson, E.J., Harris, M.P., Newell, M.A., Burthe, S.J., Reid, J.M. (2014) Site fidelity and individual variation in winter location in partially migratory European shags. *PLoS One* **9**(6), e98562. <https://doi.org/10.1371/JOURNAL.PONE.0098562>

Lebreton, J., Burnham, K.P., Clobert, J., David, R.A. (1992) Modeling survival and testing biological hypotheses using marked animals: A unified approach with case studies. *Ecol. Monogr*. **62**(1), 67–118. <https://doi.org/10.2307/2937171>

O’Brien, S., Robert, B., Tiandry, H. (2005) Consequences of violating the recapture duration assumption of mark-recapture models: A test using simulated and empirical data from an endangered tortoise population. *J. Appl. Ecol*. **42**(6), 1096–1104. <https://doi.org/10.1111/j.1365-2664.2005.01084.x>

Ugland, C.R., Acker, P., Burthe, S.J, Fortuna, R., Gunn, C., Haaland, T.R., Harris, M.P., Morley, T.I., Newell, M.A., Swann, R.L., Wanless, S., Daunt, F., Reid, J.M. (2024) Early-life variation in migration is subject to strong fluctuating survival selection in a partially migratory bird. *J. Anim. Ecol.* **93**(10), 1567–1581. <https://doi.org/10.1111/1365-2656.14172>
